# Activation of GSDME by all-*trans*-retinal increases sensitivity to photoreceptor ferroptosis

**DOI:** 10.7150/ijbs.114187

**Published:** 2025-10-27

**Authors:** Bo Yang, Kunhuan Yang, Yuling Chen, Ruitong Xi, Jiahuai Han, Shiying Li, Jingmeng Chen, Yalin Wu

**Affiliations:** 1 Xiamen Eye Center and Eye Institute of Xiamen University, School of Medicine, Xiamen University, Xiamen, Fujian 361003, China; 2 Fujian Provincial Key Laboratory of Ophthalmology and Visual Science, Fujian Engineering and Research Center of Eye Regenerative Medicine, Eye Institute of Xiamen University, School of Medicine, Xiamen University, Xiamen, Fujian, 361102, China; 3 State Key Laboratory of Cellular Stress Biology, Innovation Center for Cell Biology, School of Life Sciences, Xiamen University, Xiamen, Fujian, 361102, China; 4 Department of Ophthalmology, the First Affiliated Hospital of Xiamen University, School of Medicine, Xiamen University, Xiamen, Fujian, 361003, China; 5 School of Medicine, Xiamen University, Xiamen, Fujian, 361102, China; 6 Shenzhen Research Institute of Xiamen University, Shenzhen, Guangdong, 518063, China

**Keywords:** GSDME, photoreceptor, ferroptosis, macular degeneration, all-*trans*-retinal

## Abstract

Impaired clearance of all-*trans*-retinal (atRAL) due to visual cycle dysfunction contributes to photoreceptor atrophy, a key pathological hallmark of Stargardt disease type 1 (STGD1) and dry age-related macular degeneration (AMD). Prior studies have shown that light-induced atRAL accumulation promotes ferroptosis and activates gasdermin E (GSDME) in retinal photoreceptors of *Abca4^-/-^Rdh8^-/-^* mice, a model for STGD1 and dry AMD that exhibits visual cycle disorders. However, the role of GSDME in photoreceptor ferroptosis remains unclear. In this study, we revealed that GSDME activation by atRAL triggered photoreceptor ferroptosis and retinal atrophy via mitochondrial damage and oxidative stress. Knocking out GSDME significantly attenuated light-induced photoreceptor ferroptosis and retinal degeneration in *Abca4^-/-^Rdh8^-/-^* mice. Moreover, deleting the *Gsdme* gene in photoreceptor cells prevented atRAL-induced ferroptosis by inhibiting mitochondrial reactive oxygen species (mitoROS) production, iron overload, and lipid peroxidation. Notably, treatment with the mitoROS scavenger MitoTEMPO mitigated ferroptosis in atRAL-loaded photoreceptor cells and dramatically relieved photoreceptor ferroptosis and retinal degeneration in light-exposed *Abca4^-/-^Rdh8^-/-^* mice. We found that both GSDME elimination and MitoTEMPO treatment repressed atRAL-induced photoreceptor ferroptosis and retinal atrophy by inactivating the mitoROS-induced oxidative stress. In conclusion, GSDME-mediated photoreceptor ferroptosis is crucial for inducing structural and functional damage of the retina in retinopathies caused by atRAL accumulation, thereby providing new therapeutic insights for the prevention and treatment of STGD1 and dry AMD.

## Introduction

The metabolism of retinoids is critical for maintaining the integrity of the visual cycle, which is also essential for retinal health^1^. The visual cycle begins when a photon interacts with rhodopsin within the disk membrane of photoreceptor outer segment (POS)^2^,^3^. Photobleaching of rhodopsin releases all-*trans*-retinal (atRAL)^2^,^3^. The ABC transporter 4 (ABCA4) translocates atRAL to the cytoplasm of POS where it is reduced to vitamin A under the catalysis of retinol dehydrogenase 8 (RDH8)^4^-^6^. Thus, ABCA4 and RDH8 play important roles in facilitating the clearance of free atRAL, which is crucial for maintaining photoreceptor homeostasis. Dysregulation of this process leads to the accumulation of atRAL in photoreceptors and the retinal pigment epithelium (RPE), contributing to the pathogenesis of Stargardt disease type 1 (STGD1) and dry age-related macular degeneration (AMD)^7^,^8^. Mice lacking the *Abca4* and *Rdh8* genes (*Abca4^-/-^Rdh8^-/-^* mice) exhibit defects in atRAL clearance and develop phenotypes similar to those of STGD1 and dry AMD, including photoreceptor atrophy and RPE degeneration^9^,^10^. These pieces of evidence suggest a direct link between atRAL toxicity and retinal degeneration.

Ferroptosis, a programmed cell death, is characterized by iron overload and lipid peroxidation^11^,^12^. Recently, numerous studies have shown that ferroptosis plays a significant role in diverse pathophysiological conditions, including cardiovascular and neurodegenerative diseases^13^-^15^. Our previous studies have also demonstrated that photoreceptor degeneration caused by atRAL involves ferroptosis induced by heme oxygenase-1 (HO-1)^16^,^17^. HO-1 is a stress-inducible isoenzyme, which catalyzes the conversion of heme into biliverdin, carbon monoxide, and ferrous iron (Fe^2+^)^18^. HO-1 is largely regulated by the Kelch-like ECH-associated protein 1 (KEAP1)/nuclear factor-erythroid 2-related factor-2 (NRF2) signaling pathway^19^. In the resting state, KEAP1 binds to NRF2 and promotes its degradation. Oxidative stress induces the dissociation of KEAP1 from NRF2, allowing NRF2 to enter the nucleus to facilitate the expression of HO-1^20^,^21^. HO-1 increases Fe^2+^ levels by decomposing heme, which in turn aggravates oxidative stress through the Fenton reaction and finally triggers ferroptosis^22^,^23^. These lines of investigation demonstrate that the KEAP1/NRF2/HO-1 signaling pathway plays a key role in ferroptosis.

Gasdermins (GSDMs) are a family of pore-forming effector proteins, such as GSDMD and GSDME, which facilitate membrane permeabilization^24^,^25^. In their inactive state, the pore-forming N-terminal domains of GSDMs are obscured by their C-terminal segment^26^,^27^. After the linker region separating the N and C domains is cleaved, the N-terminal fragment is liberated and then inserts and oligomerizes in lipid membranes to form pores^26^,^28^. The formation of GSDM pores in the plasma membrane causes cell swelling and necrosis, referred to as pyroptosis^25^,^29^. Additionally, the N-terminal fragments of GSDMs can permeate mitochondrial membranes, resulting in the release of mitochondrial DNA^30^,^31^. Past studies from our laboratory have revealed that photoreceptor degeneration caused by atRAL involves the activation of GSDME rather than GSDMD^32^. However, the role of GSDME in ferroptosis has not been well defined. This study aimed to clarify that GSDME activation by atRAL contributes to photoreceptor ferroptosis leading to retinal degeneration via regulating the KEAP1/NRF2/HO-1 signaling pathway, and to provide new insights into the treatment of STGD1 and dry AMD.

## Methods and Materials

### Key reagents and antibodies

The detailed information of key reagents and antibodies is shown in the Supplementary Table S1.

### Animals

All experimental protocols involving mice were approved by the Institutional Animal Care and Use Committee of Xiamen University School of Medicine (Approval number: XMULAC20200072). C57BL/6J wild-type (WT) mice were obtained from Gempharmatech Co., Ltd. *Abca4^-/-^Rdh8^-/-^* mice were derived from our previous study^9^. *Gsdme^+/-^* mice were kindly provided by the Jiahuai Han laboratory from Xiamen University. Breeding *Gsdme^+/-^* mice gave rise to *Gsdme^-/-^* mice. The latter were subsequently crossed with *Abca4^-/-^Rdh8^-/-^* mice to generate *Abca4^-/-^Rdh8^-/-^Gsdme^-/-^* mice. The genotype of *Abca4^-/-^Rdh8^-/-^Gsdme^-/-^* mice has been identified in a recent study from our laboratory^32^. For construction of light-exposed mouse models, four-week-old C57BL/6J, *Abca4^-/-^Rdh8^-/-^* and *Abca4^-/-^Rdh8^-/-^Gsdme^-/-^* mice were dark-adapted for 2 days and then exposed to white light emitting diode (LED) light (10,000 lx, 1h), followed by dark adaptation for 5 days. On the fifth day after light illumination, the mice were examined and their eyeballs were harvested for subsequent analysis. Mice in the control group were maintained under normal dark conditions for seven days without light exposure. In the case of drug administration, *Abca4^-/-^Rdh8^-/-^* mice were intraperitoneally injected with MitoTEMPO at a dose of 5 mg/kg body weight or dimethyl sulfoxide (DMSO) as the vehicle. One hour later, the mice underwent light exposure. Next, treatment with MitoTEMPO or DMSO was continued once daily for an additional four days. The mice were examined on day five following light exposure. As for intravitreal injection, mice were prepared with mydriasis using 1% tropicamide and anesthesia using 1% pentobarbital sodium, and then underwent a single intravitreal injection of either 1.5 μl of 5 mM MitoTEMPO or phosphate-buffered saline (PBS) using a microinjector under an operating microscope. After receiving light exposure, the mice were kept in the dark for 5 days. Control *Abca4^-/-^Rdh8^-/-^* mice were injected with MitoTEMPO, DMSO or PBS without exposure to light. The pupils of all light-treated mice were dilated with 1% tropicamide prior to light exposure.

### Fundus imaging and optical coherence tomography (OCT)

Mice were anesthetized with 1% pentobarbital sodium after mydriasis with 1% tropicamide. Fundus imaging and OCT examination were performed as described previously^33^.

### Electroretinography (ERG)

Mice were examined after anesthesia with isoflurane and mydriasis with tropicamide. Retinal function was assessed by full-field ERG in an animal electroretinogram system (Diagnosys LLC). Scotopic ERG was performed under dim red light, and waveforms were recorded at different stimulus intensities (0.01, 0.1, 1, and 10 cd s/m²). The amplitudes of the a- and b-waves were quantified for evaluating the retinal response to stimulation. To ensure consistency and minimize variability, all ERG recordings were conducted at the same time of day.

### Hematoxylin and Eosin (H&E) staining

Mouse eyeballs were prepared into 5 μm tissue sections. H&E staining and measurement of the photoreceptor outer nuclear layer (ONL) thickness were performed as previously described^33^.

### Generation of knockout cell lines

The 661W photoreceptor cell line was obtained from ZishiBiotech Co., Ltd. (Shanghai, China). *Gsdme^-/-^* 661W cell line was constructed using CRISPR/Cas9, as we described previously^32^.

### Cell culture

Cells were cultured in a conventional incubator (5% CO_2_, 37°C). The cell culture medium consisted of 89% Dulbecco's Modified Eagle Medium (DMEM) medium (Gibco, Beijing, China), 10% fetal bovine serum (FBS) (Wisent, QC, Canada) and 1% penicillin-streptomycin (Biosharp, Bengbu, China).

### Cell viability

WT and *Gsdme^-/-^* 661W cells were cultured overnight and then incubated with 5 μM atRAL for 6 h. Alternatively, 661W cells were pretreated with MitoTEMPO (40, 50, and 60 μM) or DMSO for 2 h, followed by incubation with 5 µM atRAL for 6 h. Cell viability was detected by an MTS assay kit as we described previously^9^,^10^.

### Quantitative real-time PCR (qRT-PCR)

Total RNA was extracted by Trizol reagent. The qRT-PCR was performed as previously described^33^. The sequences of primers were listed in the Supplementary Table S2.

### Western blotting

Protein extraction and quantification of cells and neural retina were performed as previously described^33^. The samples after RIPA lysis were centrifuged at 4°C, and the protein concentration of the supernatant was quantified using BCA reagent. The quantified protein samples were separated by SDS-PAGE, and then were transferred to a PVDF membrane in an ice-water bath. After blocking with 5% skim milk for 1 h at room temperature, incubating with primary antibody (1:1000 dilution) at 4°C for 12 h, and incubating with secondary antibody (1:2000 dilution) for 2 h at room temperature, the membrane was scanned on the imaging instrument (Bio-Rad, Canada).

### Confocal fluorescence imaging

Cells were incubated with a variety of probes at 37°C, then washed three times with PBS to remove residual probes. They were then observed under a Zeiss LSM 880 confocal microscope (Jena, Germany). The following probes were used for specific detections: CellROX™ Deep Red probe (10 μM, 30 min) for intracellular ROS detection, FerroOrange probe (1 μM, 30 min) and FeRhoNox-1 probe (5 μM, 30 min) for intracellular Fe^2+^ detection, C11-BODIPY 581/591 probe (10 μM, 40 min) for lipid peroxidation detection, Rhodamine-123 probe (10 μM, 30 min) for mitochondrial membrane potential detection, and MitoSOXTM Red probe (5 μM, 30 min) for mitoROS detection. Cell nuclei were imaged with 10 μM Hoechst 33342. Images were captured using the Zeiss LSM 880 confocal microscope (Jena, Germany).

### Separation of cytoplasmic and nuclear extracts

Cells were detached with trypsin and harvested by centrifugation at 500×g for 3 min. The supernatant was carefully aspirated and discarded, leaving a dry cell pellet. Ice-cold CER I was added to the pellet, and the mixture was vortexed vigorously to achieve complete resuspension. After incubation on ice for 10 min, ice-cold CER II was added, and the tube was vortexed vigorously again. Following a further 3 min on ice, the sample was vortexed and centrifuged at 16,000×g for 5 min. The supernatant (cytoplasmic extract) was promptly transferred to a new pre-chilled tube. The remaining insoluble pellet, containing nuclear material, was resuspended in ice-cold NER and vortexed vigorously. The sample was kept on ice and subjected to additional 15-s vortexing cycles at 10-min intervals over a period of 40 min. After a final centrifugation at 16,000×g for 10 min, the supernatant (nuclear extract) was transferred to a clean pre-chilled tube and kept on ice. All extracts were stored at -80 °C until use.

### Immunofluorescence staining

Cells, retinal sections and RPE/choroids flat mounts were first pre-processed at room temperature. Cell were fixed for 15 min with fixative solution, permeabilizated for 20 min with 0.2% Triton X-100, and then blocked for 1h with 2% bovine serum albumin (BSA). For retinal paraffin sections, the sections were dewaxed, subjected to antigen retrieval, and subsequently blocked with 2% BSA for 1 h. For the RPE/choroids flat mounts, mouse eyeballs were fixed in the fixative solution for 30 min at room temperature, and RPE/choroids flat mounts were carefully collected. Subsequently, the flat mounts were permeabilizated for 30 min with 1% Triton X-100, and then blocked for 1 h with 5% BSA containing 0.3% Triton X-100. The BSA-blocked samples incubated with primary antibodies (1:200 dilution) at 4°C overnight, followed by 2 h of exposure to fluorescent secondary antibodies (1:200 dilution) at room temperature. The nuclei of samples were stained with DAPI. Images were taken with Zeiss LSM 880 confocal microscope.

### Co-immunoprecipitation

Co-immunoprecipitation was performed using the Pierce Crosslink Magnetic IP/Co-IP Kit. After washing with cross-linking buffer, the magnetic beads were incubated with the antibody for 15 minutes. The beads were then washed three times with cross-linking buffer to remove unbound antibodies. Next, the magnetic beads were cross-linked with the antibody using disuccinimidyl suberate (DSS) for 30 minutes, followed by two washes with immunoprecipitation lysis buffer. The antibody-crosslinked magnetic beads were then incubated with cell lysate for 2 hours at room temperature, and then washed twice with immunoprecipitation lysis buffer. Finally, the antigen bound to the magnetic beads was eluted with elution buffer and detected by western blotting.

### Detection of citric acid and α-ketoglutarate

The levels of citric acid and α-ketoglutarate in the neural retina were detected using the Amplex Red Citrate Assay Kit and the Amplex Red α-Ketoglutarate Assay Kit, respectively. Briefly, the neural retina was cryogenically ground in lysis buffer, then centrifuged at 12,000 rpm for 5 minutes at 4ºC. The supernatant was collected for detection. The relative content was calculated based on the standard curve provided by the kit.

### Bioinformatics analysis

Bioinformatics analysis was conducted using R (version 4.4.1) within the RStudio environment (version 2023.06.0). The GSE29801 dataset was extracted from the Gene Expression Omnibus (GEO) database and subjected to quality control. Gene expression data for the neural retina were sorted into two groups: the geographic atrophy group (GSM738672, GSM738702, GSM738711) and the age-matched control group (GSM738642, GSM738645, GSM738657). The pheatmap package (Version 1.0.12) was used to generate a heatmap of iron homeostasis-related genes. Additionally, gene expression data were analyzed for enrichment of iron homeostasis-related gene sets ('HP_ABNORMALITY_OF_IRON_HOMEOSTASIS' and 'GOBP_INTRACELLULAR_IRON_ION_HOMEOSTASIS') using the Gene Set Enrichment Analysis (GSEA) software (Version 4.3.3).

### Statistical analysis

Results are exhibited as the mean ± standard deviation (SD) from at least three independent experiments. Statistical analyses were performed in GraphPad Prism software (version 8.0) using one-way or two-way analysis of variance (ANOVA) followed by Tukey's multiple comparison tests. The level of statistical significance was set at *p*-value < 0.05.

## Results

### Loss of the *Gsdme* gene protects against photoinduced retinal degeneration and photoreceptor ferroptosis in *Abca4^-/-^Rdh8^-/-^* mice

**Figure 1:**
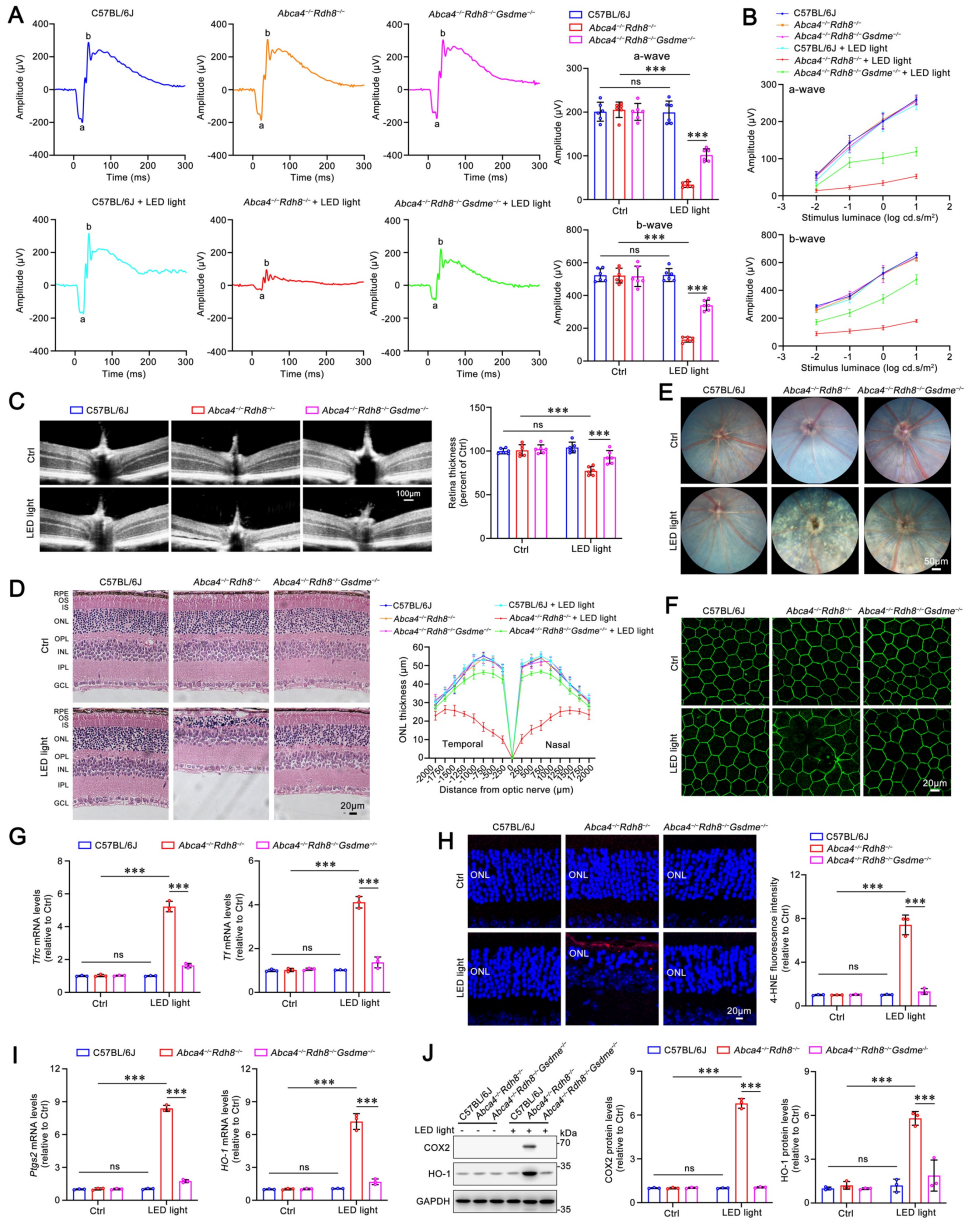
** Gene deletion of GSDME mitigates light-induced retinal degeneration and photoreceptor ferroptosis in* Abca4^-/-^Rdh8^-/-^* mice.** (A) Full-flash ERG, 1 cd s/m^2^ (n=6). (B) Full-flash ERG with stimulus luminance levels of 0.01, 0.1, 1 and 10 cd s/m^2^ (n=6). (C) Retinal thickness was examined by OCT (n=6). Scale bars, 100 μm. (D) ONL thickness was measured by H&E staining (n=6). Scale bars, 20 μm. (E) Fundus imaging. Scale bars, 50 μm. (F) Whole-mount immunofluorescence staining for the tight junction protein ZO-1 (*green*). Scale bars, 20 μm. (G) The mRNA levels of *Tfrc* and *Tf* were analyzed by qRT-PCR in the neural retina (n=3). (H) 4-HNE levels were quantified by immunofluorescence in the neural retina (n=3). Scale bars, 20 μm. (I) The mRNA levels of *Ptgs2* and *HO-1* were analyzed by qRT-PCR in the neural retina (n=3). (J) The protein levels of COX2 and HO-1 were quantified by immunoblotting in the neural retina (n=3). ns, not significant. ***p < 0.001.

Previous evidence has indicated that, compared with light-exposed and light-free C57BL/6J mice as well as light-free *Abca4**^-/-^**Rdh8**^-/-^*** mice, *Abca4^-/-^Rdh8*^*-/-*^ mice upon exposure to light exhibit a rapid and excess accumulation of atRAL in the retina, which leads to retinal degeneration^9^,^10^. Our prior report has demonstrated that GSDME activation, which is reflected by increased protein levels of GSDME-N, is observed in the photoreceptors of *Abca4^-/-^Rdh8^-/-^* mice exposed to light^32^. Dark-adapted mice were exposed for 1 h to LED light following pupil dilation, and then returned to dark conditions and fed for 5 days. Photoreceptor responses to light stimuli were assessed using full-field ERG. The data revealed that knockout of the *Gsdme* gene in *Abca4^-/-^Rdh8^-/-^* mice notably prevented the light-induced reduction in a- and b-wave amplitudes, suggesting a protective role of GSDME deficiency in preserving retinal function (**Figure 1A-1B**). Retinal OCT and H&E staining analyses showed that *Gsdme* gene knockout effectively mitigated light-induced thinning in the neural retina and photoreceptor ONL thickness of *Abca4^-/-^Rdh8^-/-^* mice (**Figure 1C-1D**). Moreover, GSDME deficiency also alleviated RPE degeneration in light-exposed *Abca4^-/-^Rdh8^-/-^* mice (**Figure 1E-1F**). In comparison with *Abca4^-/-^Rdh8^-/-^* mice after light exposure,* Abca4^-/-^Rdh8^-/-^Gsdme^-/-^* mice following light illumination exhibited fewer punctate lesions in the fundus (**Figure 1E**), and showed a reduced disruption of RPE tight junctions, as indicated by ZO-1 staining (**Figure 1F**).

To evaluate the impact of GSDME on ferroptosis in the neural retina of light-exposed *Abca4^-/-^Rdh8^-/-^* mice, we measured the expression of genes related to iron homeostasis and assessed lipid peroxidation levels. Transferrin (TF) and transferrin receptor (TFRC) play critical roles in maintaining cellular iron homeostasis by facilitating the transfer of Fe^3+^ from the extracellular space into cells, where it is subsequently reduced to Fe^2+34,35^. Increased levels of TF and TFRC are indicative of intracellular iron overload^36^. Following *Gsdme* gene deletion, the mRNA expression of *Tf* and *Tfrc* in the neural retina of *Abca4^-/-^Rdh8^-/-^* mice after light exposure was significantly downregulated (**Figure 1G**), thereby suggesting that iron overload is suppressed. The results of immunofluorescence staining also demonstrated that knocking out* Gsdme* gene significantly reduced the levels of 4-HNE (**Figure 1H**), a maker of lipid peroxidation^37^. In addition, the elimination of the *Gsdme* gene remarkably decreased the gene and protein levels of COX2 and HO-1, two well-known ferroptosis biomarkers^22^,^38^, in the neural retina of light-exposed *Abca4^-/-^Rdh8^-/-^* mice (**Figure 1I-1J**). Collectively, these findings imply that *Gsdme* gene deficiency prevents retinal function decline and ameliorates light-induced photoreceptor ferroptosis and retinal degeneration in *Abca4^-/-^Rdh8^-/-^* mice by alleviating iron overload and lipid peroxidation.

### GSDME activation by atRAL promotes 661W photoreceptor cell ferroptosis through iron dyshomeostasis and lipid peroxidation

**Figure 2:**
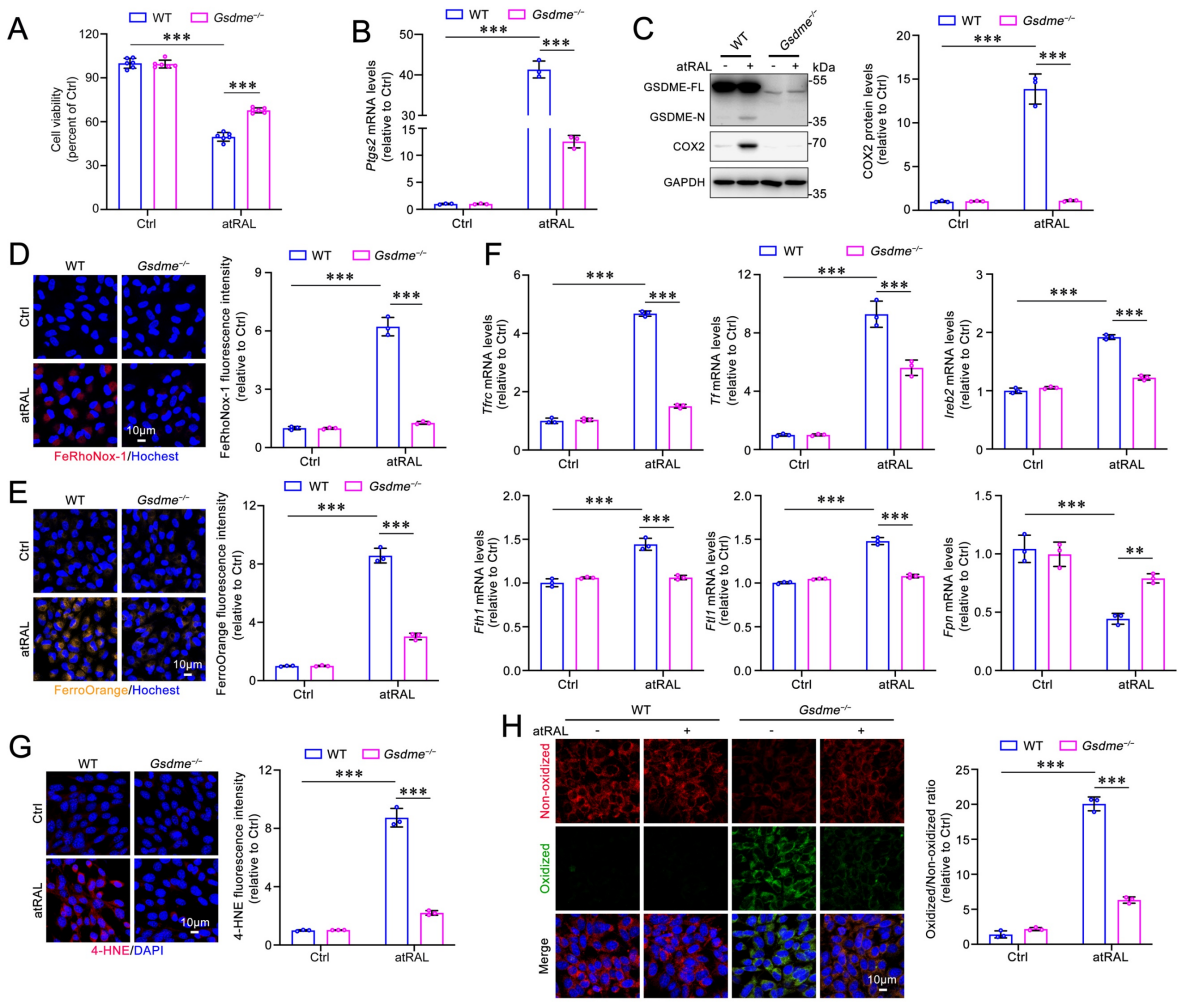
**Gene knockout of GSDME alleviates iron dyshomeostasis and lipid peroxidation in atRAL-loaded 661W cells.** (A) Cell viability. (B) The mRNA levels of *Ptgs2* were analyzed by qRT-PCR (n=3). (C) The protein levels of GSDME-FL, GSDME-N and COX2 were examined by immunoblotting (n=3). Intracellular Fe^2+^ was assessed by (D) FeRhoNox-1 staining (n=3) and (E) FerroOrange staining (n=3), respectively. (F) The mRNA levels of iron homeostasis-related genes (*Tfrc*, *Tf*, *Ireb2*, *Fth1*, *Ftl1* and *Fpn*) were analyzed by qRT-PCR (n=3). (G) 4-HNE levels were quantified by immunofluorescence (n=3). (H) Lipid peroxidation was assessed by an image-iT^TM^ Lipid Peroxidation Kit (n=3). Scale bars (D, E, G and H), 10 μm. **p < 0.01 and ***p < 0.001.

Murine photoreceptor cell line 661W was utilized as an in vitro model to investigate the role of GSDME in photoreceptor ferroptosis caused by atRAL. WT or *Gsdme* gene knockout (*Gsdme^-/-^*) 661W cells were exposed to atRAL for 6 h. Deletion of the *Gsdme* gene remarkably enhanced the survival of 661W cells following atRAL exposure (**Figure 2A**). The results of qRT-PCR and western blotting showed that the deletion of GSDME significantly decreased mRNA and protein levels of ferroptosis biomarker COX2 encoded by the *Ptgs2* gene in atRAL-loaded 661W cells (**Figure 2B-2C**). Intracellular Fe^2+^ levels were assessed using fluorescent probes FeRhoNox-1 and FerroOrange. The fluorescence imaging revealed that the deletion of the* Gsdme* gene significantly alleviated iron overload in atRAL-loaded 661W cells (**Figure 2D-2E**). To assess the impact of GSDME on iron homeostasis in photoreceptor cells exposed to atRAL, we conducted qRT-PCR analysis of genes involved in iron metabolism^16^, including *Tfrc*, *Tf*, *iron-responsive element binding protein 2* (*Ireb2*), *ferritin heavy chain 1* (*Fth1*), *ferritin light chain 1* (*Ftl1*), and *ferroportin* (*Fpn*). The data manifested that in atRAL-treated *Gsdme^-/-^* 661W cells, the mRNA levels of these iron homeostasis-related genes were significantly restored compared to those in atRAL-loaded WT 661W cells (**Figure 2F**). Moreover, fluorescence imaging of 4-HNE and C11-BODIPY, as well as flow cytometric quantification of C11-BODIPY, revealed that *Gsdme* gene knockout effectively mitigated lipid peroxidation in atRAL-exposed 661W cells (**Figure 2G-2H** and **Figure S1A**). These findings suggest that genetic deletion of the *Gsdme* gene mitigates atRAL-driven photoreceptor cell ferroptosis by reducing iron overload and lipid peroxidation.

### Gene knockout of *Gsdme* attenuates mitochondrial damage and inhibits the KEAP1/NRF2/HO-1 signaling pathway in atRAL-loaded 661W cells

**Figure 3:**
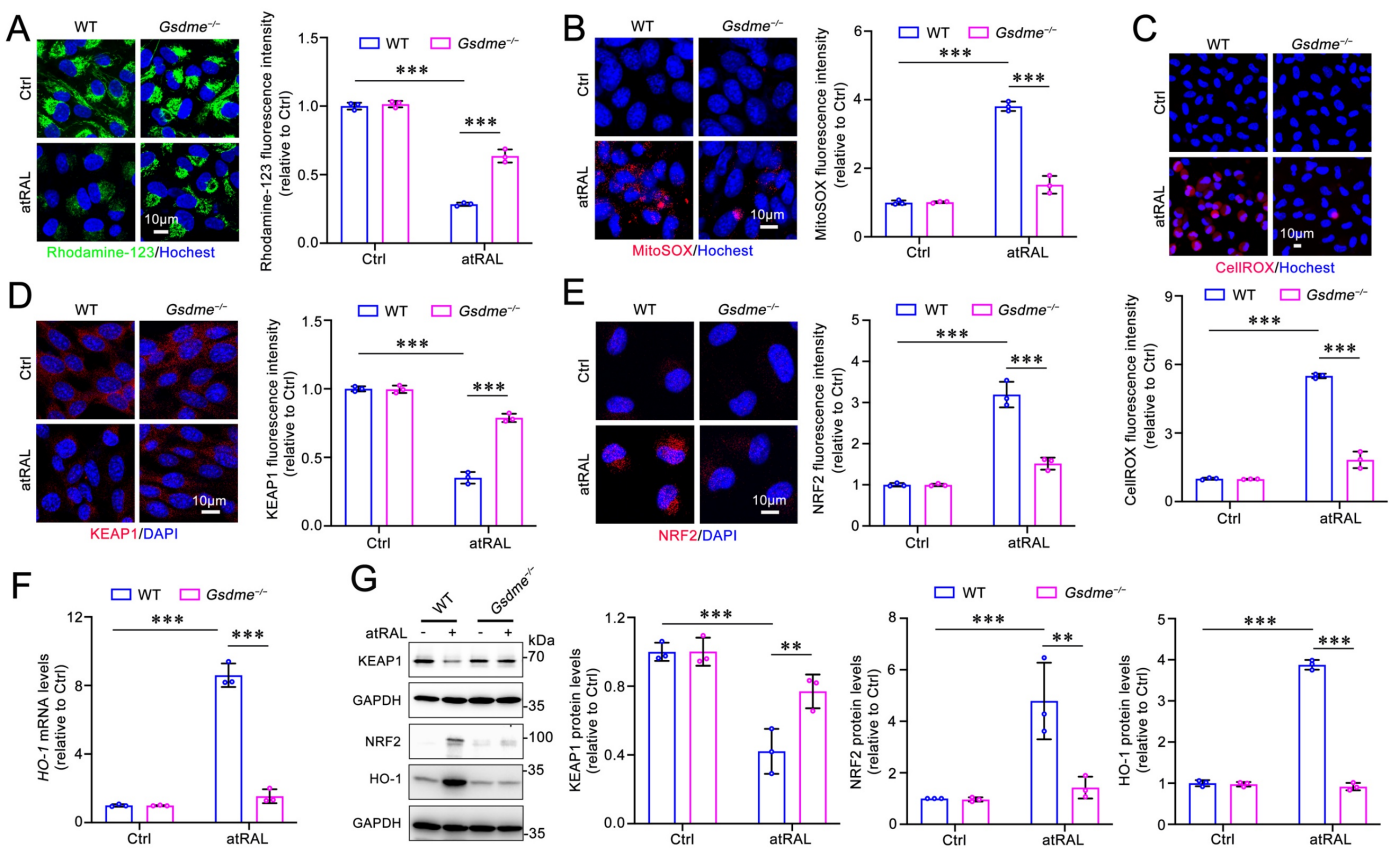
** Elimination of GSDME ameliorates mitochondrial damage and the KEAP1/NRF2/HO-1 signaling in atRAL-loaded 661W cells.** (A) Mitochondrial membrane potential was assessed by Rhodamine-123 staining (n=3). (B) MitoROS levels were examined by MitoSOX^TM^ Red staining (n=3). (C) ROS generation was visualized with CellROX^TM^ Deep Red staining (n=3). The expressions of (D) KEAP1 (n=3) and (E) NRF2 (n=3) were quantified by immunofluorescence. (F) The mRNA levels of *HO-1* were analyzed by qRT-PCR (n=3). (G) The protein levels of KEAP1, NRF2 and HO-1 were examined by immunoblotting (n=3). Scale bars (A-E), 10 μm. **p < 0.01 and ***p < 0.001.

Our previous studies have demonstrated that photoreceptor ferroptosis induced by atRAL involves mitochondrial injury and the activation of the KEAP1/NRF2/HO-1 signaling pathway^17^. Due to the ability of GSDME to damage the mitochondria by forming pores via its N-terminal domain^32^, we assessed its impact on mitochondrial membrane potential and mitochondrial reactive oxygen species (mitoROS) levels using fluorescent probes Rhodamine-123 and MitoSOX^TM^ Red in atRAL-treated *Gsdme^-/-^* 661W cells, respectively. The fluorescence imaging revealed that *Gsdme* gene knockout visibly restored the mitochondrial membrane potential and reduced mitoROS levels in 661W cells loaded with atRAL (**Figure 3A-3B**). There is already evidence that excessive intracellular ROS is capable of activating the KEAP1/NRF2/HO-1 signaling pathway^17^. Confocal imaging using the probe CellROX^TM^ Deep Red reflected that deleting* Gsdme* significantly attenuated intracellular ROS production in atRAL-treated 661W cells (**Figure 3C**). In addition, eliminating the *Gsdme* gene in atRAL-loaded 661W cells resulted in increased KEAP1 protein levels, decreased NRF2 protein levels, and reduced mRNA and protein levels of HO-1 (**Figure 3D-3G**). These results suggest that the activation of GSDME by atRAL promotes photoreceptor cell ferroptosis via triggering mitochondrial impairment and activating the KEAP1/NRF2/HO-1 axis.

### MitoTEMPO protects 661W cells from atRAL-induced ferroptosis via repressing the mitoROS-driven KEAP1/NRF2/HO-1 signaling pathway

**Figure 4:**
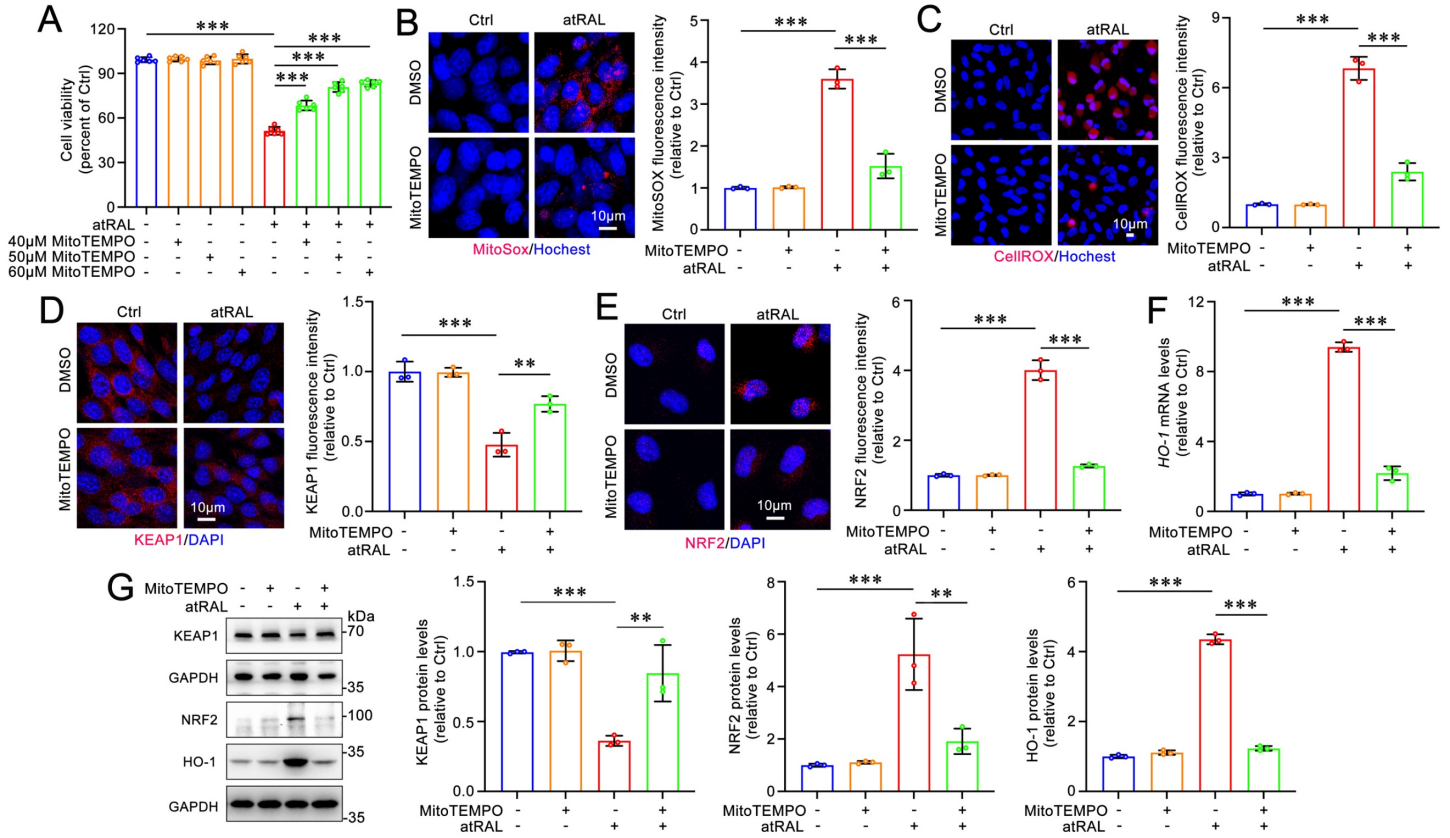
** MitoTEMPO prevents the activation of the KEAP1/NRF2/HO-1 signaling pathway in atRAL-loaded 661W cells by reducing the production of mitoROS.** (A) Cell viability. 661W cells were pretreated with MitoTEMPO at gradient concentrations of 40, 50 and 60 μM for 2 h and then exposed to 5 μM atRAL for 6 h. (B) Mitochondrial superoxide levels were examined by MitoSOX^TM^ Red staining (n=3). (C) The production of ROS was detected by CellROX^TM^ Deep Red staining (n=3). The expression of (D) KEAP1 (n=3) and (E) NRF2 (n=3) were quantified by immunofluorescence. (F) The mRNA levels of *HO-1* were analyzed by qRT-PCR (n=3). (G) Western blots and quantification of KEAP1, NRF2 and HO-1 (n=3). Scale bars (B-E), 10 μm. **p < 0.01 and ***p < 0.001.

To confirm the regulatory effect of mitoROS on the KEAP1/NRF2/HO-1 signaling pathway in atRAL-loaded 661W cells, MitoTEMPO, a potent mitoROS scavenger^39^, was used. The MTS assay results showed that treatment with MitoTEMPO at concentrations of 40, 50 and 60 μM significantly and concentration-dependently enhanced the viability of atRAL-treated 661W cells (**Figure 4A**). Subsequent experiments were conducted using 50 μM MitoTEMPO. The fluorescence imaging with probes MitoSOX^TM^ Red and CellROX^TM^ Deep Red revealed that MitoTEMPO significantly inhibited the generation of mitoROS and intracellular ROS in 661W cells exposed to atRAL (**Figure 4B-4C**). As expected, after treatment with MitoTEMPO, atRAL-loaded 661W cells visibly exhibited increased protein levels of KEAP1, decreased protein levels of NRF2, and reduced mRNA and protein levels of HO-1 (**Figure 4D-4G**).

**Figure 5:**
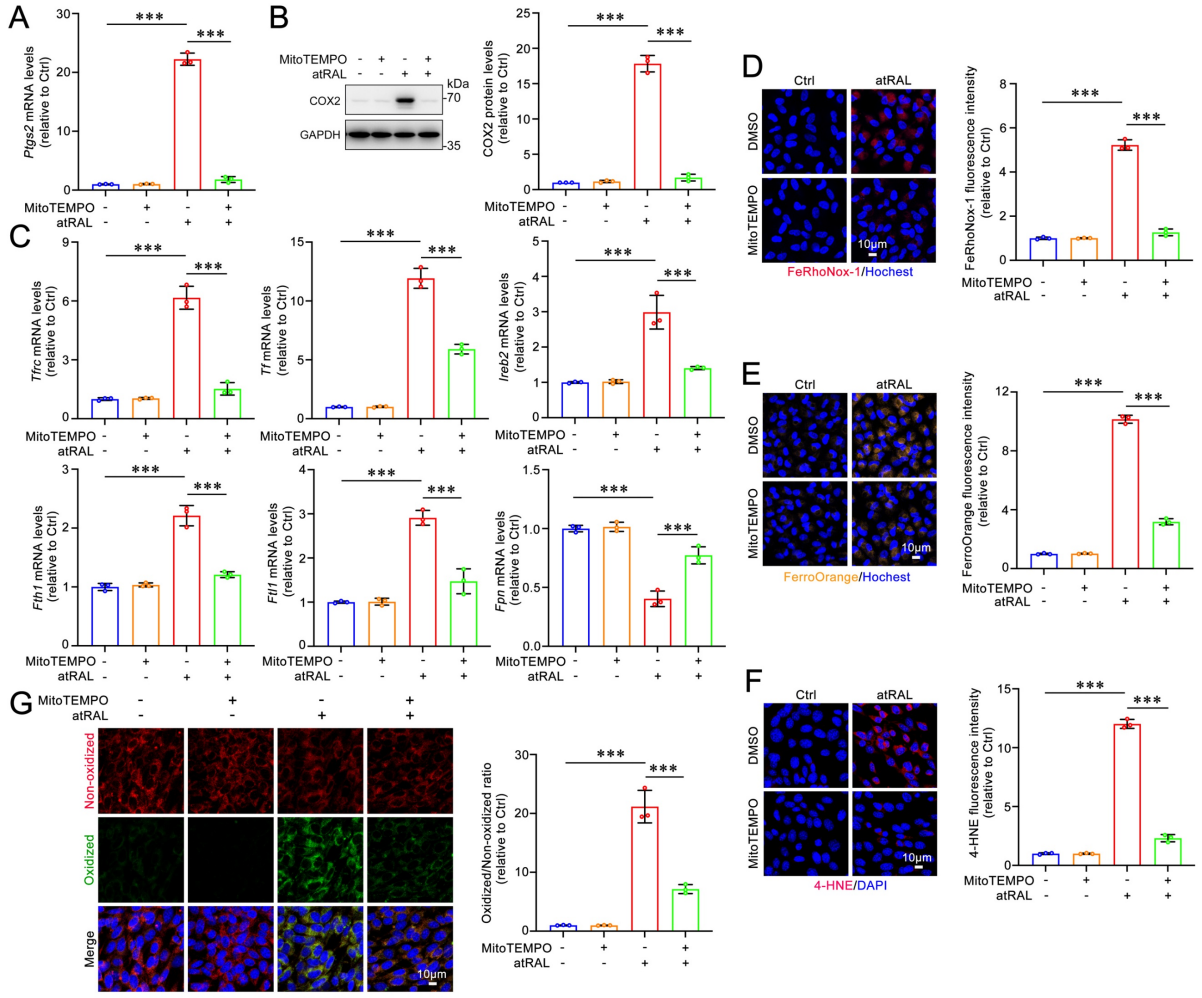
**MitoTEMPO attenuates iron dyshomeostasis and lipid peroxidation in atRAL-loaded 661W cells.** (A) Quantification of the mRNA levels of *Ptgs2* encoding COX2 (n=3). (B) The protein levels of COX2 was examined by immunoblotting (n=3). (C) Quantification of the mRNA levels of iron homeostasis-related genes (*Tfrc*, *Tf*, *Ireb2*, *Fth1*, *Ftl1* and *Fpn*) (n=3). Intracellular levels of Fe^2+^ was evaluated using (D) FeRhoNox-1 staining (n=3) and (E) FerroOrange staining (n=3), respectively. (F) 4-HNE levels were quantified by immunofluorescence (n=3). (G) Lipid peroxidation levels were assessed by live cell imaging (n=3). Scale bars (D-G), 10 μm. ***p < 0.001.

On the other hand, MitoTEMPO treatment significantly decreased the gene and protein levels of the ferroptosis biomarker COX2 in atRAL-loaded 661W cells (**Figure 5A-5B**). The qRT-PCR analysis of iron homeostasis-related genes revealed that MitoTEMPO obviously restored the mRNA levels of *Tfrc*, *Tf*, *Ireb2*, *Fth1*,* Ftl1* and *Fpn* in 661W cells exposed to atRAL (**Figure 5C**). Fluorescence imaging using FeRhoNox-1 and FerroOrange probes showed that MitoTEMPO dramatically prevented the atRAL-induced increase of Fe^2+^ levels in 661W cells (**Figure 5D-5E**). Moreover, MitoTEMPO remarkably ameliorated atRAL-induced lipid peroxidation in 661W cells (**Figure 5F-5G** and **Figure S1B**). Evidence from several studies have indicated that the activation of the KEAP1/NRF2/HO-1 signaling pathway is closely associated with ferroptotic cell death^16^-^18^,^22^,^23^. These findings suggest that the production of mitoROS induced by atRAL evokes photoreceptor cell ferroptosis via activating the KEAP1/NRF2/HO-1 signaling pathway.

### MitoTEMPO mitigates light-induced retinal degeneration and photoreceptor ferroptosis in *Abca4^-/-^Rdh8^-/-^* mice via inactivating the KEAP1/NRF2/HO-1 signaling pathway

**Figure 6:**
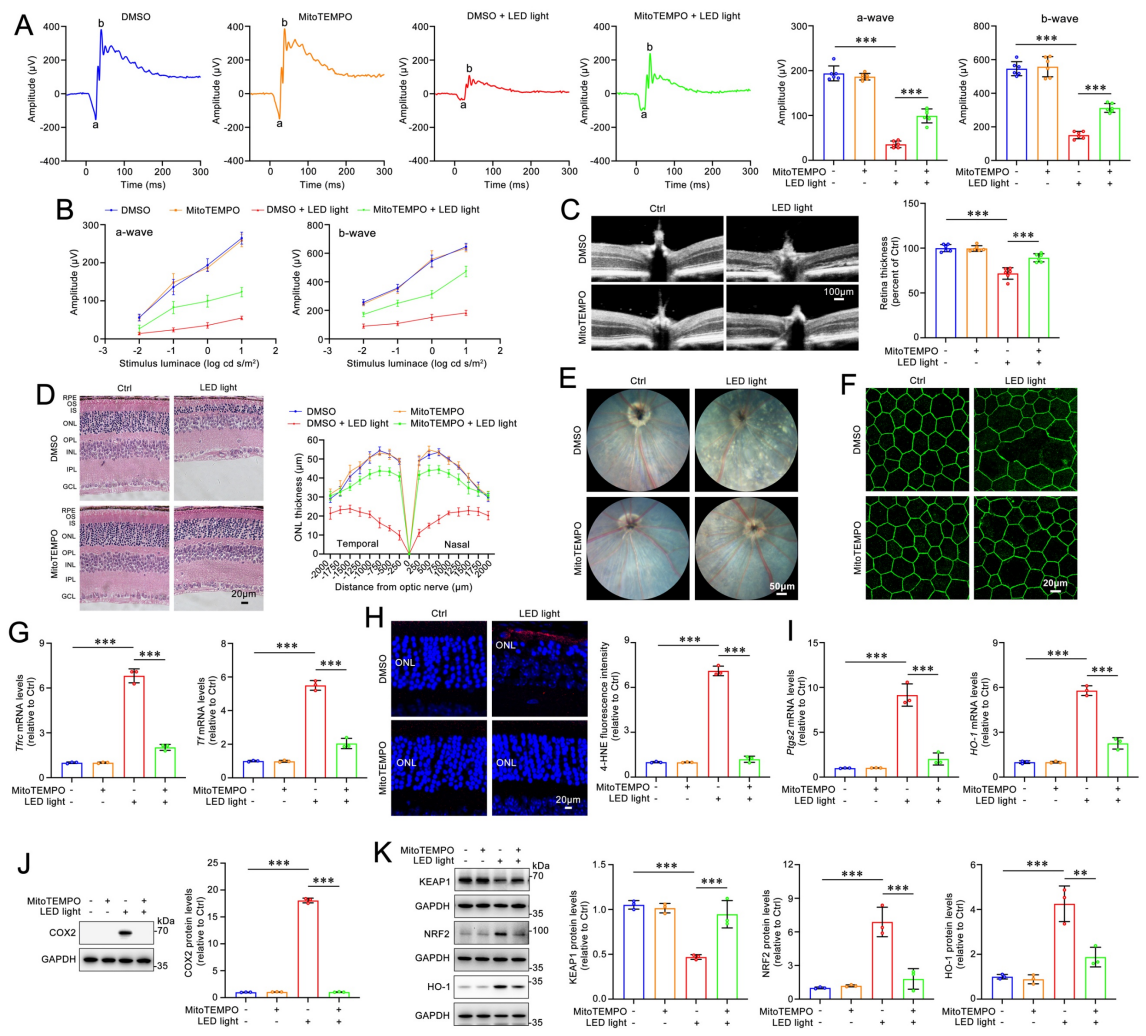
**MitoTEMPO relieves retinal degeneration and photoreceptor ferroptosis in light-exposed** ***Abca4******^-/-^Rdh8^-/-^* mice.** (A) Full-flash ERG, 1 cd s/m^2^ (n=6). (B) Full-flash ERG with stimulus luminance levels of 0.01, 0.1, 1 and 10 cd s/m^2^ (n=6). (C) Retinal thickness was examined by OCT (n=6). Scale bars, 100 μm. (D) ONL thickness was measured by H&E staining (n=6). Scale bars, 20 μm. (E) Fundus imaging. Scale bars, 50 μm. (F) Whole-mount immunofluorescence staining for the tight junction protein ZO-1 (*green*). Scale bars, 20 μm. (G) The mRNA levels of *Tfrc* and *Tf* were analyzed by qRT-PCR in the neural retina (n=3). (H) 4-HNE levels were quantified by immunofluorescence in the neural retina (n=3). Scale bars, 20 μm. (I) The mRNA levels of *Ptgs2* and *HO-1* were analyzed by qRT-PCR in the neural retina (n=3). The protein levels of (J) COX2 (n=3), and (K) KEAP1, NRF2 and HO-1 (n=3) were quantified by immunoblotting in the neural retina (n=3). **p < 0.01 and ***p < 0.001.

Dark-adapted* Abca4^-/-^Rdh8^-/-^* mice were administered MitoTEMPO (5 mg/kg) or DMSO intraperitoneally 1 h prior to light exposure. The mice were subsequently exposed to LED light for 1 h following pupil dilation. Next, the once-daily MitoTEMPO treatment was continued for an additional four days. The ERG results demonstrated that MitoTEMPO effectively prevented the light-induced reduction in a- and b-wave amplitudes in *Abca4^-/-^Rdh8^-/-^* mice, indicating a significant improvement in retinal function (**Figure 6A-6B**). The results of retinal OCT and H&E staining showed that intraperitoneal administration of MitoTEMPO significantly mitigated neuroretinal injury and prevented the decrease in thickness of the retina and ONL in *Abca4^-/-^Rdh8^-/-^* mice following light exposure (**Figure 6C-6D**). In addition, retinal fundus imaging revealed that intraperitoneally administered MitoTEMPO markedly alleviated light-induced RPE degeneration in *Abca4^-/-^Rdh8^-/-^* mice (**Figure 6E**). Immunofluorescence staining for ZO-1 demonstrated that MitoTEMPO significantly preserved the integrity of RPE tight junctions in light-exposed *Abca4^-/-^Rdh8^-/-^* mice (**Figure 6F**). Moreover, intraperitoneal administration of MitoTEMPO significantly reduced the mRNA levels of *Tfrc* and *Tf*, as well as the levels of the lipid peroxidation marker 4-HNE in the neural retina of *Abca4^-/-^Rdh8^-/-^* mice with light exposure (**Figure 6G-6H**). As expected, intraperitoneally injected MitoTEMPO clearly reduced the mRNA and protein levels of HO-1 and COX2, decreased protein levels of NRF2, and elevated protein levels of KEAP1 in the neural retina of light-exposed *Abca4^-/-^Rdh8^-/-^* mice (**Figure 6I-6K**). Overall, these lines of evidence suggest the ability of MitoTEMPO to prevent retinal degeneration and photoreceptor ferroptosis in mice with impaired atRAL clearance through inhibiting the activation of the KEAP1/NRF2/HO-1 signaling pathway.

## Discussion

This study provided evidence for the involvement of GSDME in photoreceptor ferroptosis caused by atRAL, and presented several significant findings. First, GSDME deletion mitigates retinal degeneration in mice with defects in atRAL clearance. Second, the elimination of GSDME attenuates iron dyshomeostasis and lipid peroxidation in the photoreceptors accumulating atRAL. Third, GSDME activation by atRAL stimulates photoreceptor ferroptosis by mitoROS-induced activation of the KEAP1/NRF2/HO-1 signaling pathway. Finally, MitoTEMPO treatment ameliorates retinal damage in mice with compromised clearance of atRAL.

The GSDMs represents a class of pore-forming proteins^24^. Although the role of GSDMs in pyroptosis has been extensively investigated^25^, information regarding their relationship to ferroptosis remains very limited. We have previously shown that photoreceptor damage by atRAL implicates the activation of GSDME rather than GSDMD^32^. To further confirm the status of GSDME activation in neural retina of light-exposed *Abca4^-/-^Rdh8^-/-^* mice, four-week-old C57BL/6J and* Abca4^-/-^Rdh8^-/-^* mice that underwent light exposure were reared in the dark for 1, 3 and 5 days, followed by examination of the retina using co-immunofluorescence staining for GSDME-N and RPE65, a marker of the RPE layer. Control C57BL/6J and *Abca4^-/-^Rdh8^-/-^* mice were kept normally for 7 days in the dark without exposure to light. Compared to control C57BL/6J mice, GSDME activation was significantly induced and increased over time in photoreceptor ONL of *Abca4^-/-^Rdh8^-/-^* mice, but it did not occur in neural retina of light-exposed C57BL/6J mice and control *Abca4^-/-^Rdh8^-/-^* mice (**Figure S2**). Herein, it was observed that GSDME deletion ameliorated photoreceptor ferroptosis and retinal degeneration induced by atRAL (**Figure 1** and **Figure 2**), thereby suggesting a link between GSDME and photoreceptor ferroptosis. A previous study from our laboratory has indicated that photoreceptor ferroptosis induced by atRAL involves HO-1 activation^17^. In the present study, we found that GSDME deletion increased the resistance of atRAL-accumulating photoreceptors to ferroptosis, at least in part, via inhibiting the mitoROS/KEAP1/NRF2/HO-1 signaling pathway (**Figure 1**,** Figure 3** and **Figure S3**). Given that the current findings suggested that GSDME played a regulatory role in ferroptosis, we further evaluated whether GSDME-N interacted with key proteins involved in ferroptosis (GPX4, HO-1, KEAP1, and NRF2) in atRAL-loaded 661W cells. However, co-immunoprecipitation results showed that GSDME-N did not directly bind to them (**Figure S4**).

Indeed, scavenging mitoROS with MitoTEMPO prevented the KEAP1/NRF2/HO-1 signaling pathway activation and ameliorated iron dyshomeostasis and lipid peroxidation in atRAL-loaded 661W cells and the neural retina of light-exposed *Abca4^-/-^Rdh8^-/-^* mice (**Figure 4**, **Figure 5** and **Figure 6**). Similar to the efficacy observed with intraperitoneal injection (**Figure 6**), a single intravitreal injection of MitoTEMPO was also effective in alleviating light-induced retinal degeneration and photoreceptor ferroptosis in *Abca4^-/-^Rdh8^-/-^* mice (**Figure S5**). Moreover, the potential systemic toxicity of MitoTEMPO was evaluated in *Abca4^-/-^Rdh8^-/-^* mice following intraperitoneal injection. The results showed that once-daily intraperitoneal injection of MitoTEMPO for 5 consecutive days did not cause damage to the retina (**Figure 6**) or to vital organs such as the heart, liver, spleen, lungs, and kidneys (**Figure S6**). A recent report discloses that the activation of GSDMD by ferroptosis contributes to heart failure^40^. To further explore whether photoreceptor ferroptosis caused by atRAL affects GSDME activation, 661W cells were pretreated for 2 h with ferroptosis inhibitor ferrostatin-1 (Fer-1) at a concentration of 20 μM and then exposed to 5 μM atRAL for 6 h. Immunoblotting results showed that Fer-1 significantly reduced protein levels of GSDME-N in atRAL-loaded 661W cells (**Figure S7**). Collectively, these findings suggest that GSDME activates ferroptosis, which in turn exacerbates GSDME activation in photoreceptors that accumulate atRAL.

Since mitoROS often affects cellular metabolism, we assessed the impact of mitoROS clearance on retinal glycolysis and tricarboxylic acid cycle (TCA) cycle activity as well as metabolic reprogramming. Citric acid and α-ketoglutarate are key metabolites in the TCA cycle 41,42. Our results showed that in light-exposed *Abca4^-/-^ Rdh8^-/-^* mice, the levels of citric acid and α-ketoglutarate in the neural retina were visibly reduced, but were restored by intraperitoneal injection with the mitoROS scavenger MitoTEMPO (**Figure S8A**). We further analyzed the expression of genes related to glycolysis and the TCA cycle, such as *glucose transporter 1* (*Glut1*), *lactate dehydrogenase A* (*Ldha*), *hexokinase 2* (*Hk2*), *pyruvate kinase muscle isozyme 2* (*Pkm2*), *mitochondrial pyruvate carrier 1* (*Mpc1*), *isocitrate dehydrogenase subunit alpha* (*Idh3a*) and *isocitrate dehydrogenase subunit beta* (*Idh3b*). The qRT-PCR results showed that MitoTEMPO treatment restored the expression of these genes, with the exception of *Idh3a* (**Figure S8B-S8C**). In addition, we evaluated the status of nicotinamide adenine dinucleotide (NAD) metabolism, which is closely related to mitochondrial metabolic reprogramming 43,44. The results showed that MitoTEMPO treatment restored the expression of the genes encoding sterile alpha and toll receptor motif-containing 1 (SARM1) and nicotinamide phosphoribosyltransferase (NAMPT) that involve mitochondrial metabolic reprogramming (**Figure S8D**). These findings indicate that the elimination of mitoROS preserves the function of glycolysis and the TCA cycle and improves mitochondrial metabolic reprogramming in the neural retina of light-exposed *Abca4^-/-^ Rdh8^-/-^* mice.

To ascertain whether GSDME-mediated mitoROS production regulates immune responses in the neural retina of *Abca4^-/-^ Rdh8^-/-^* mice following light exposure, we examined the effect of scavenging mitoROS on the expression of inflammatory cytokines and complement system components in the retinal microenvironment. The qRT-PCR analysis showed that intraperitoneally injected MitoTEMPO obviously restored the mRNA levels of inflammatory cytokines and complement system components, including *interleukin 6* (*IL6*), *tumor necrosis factor* (*Tnf*), *C-X-C motif chemokine ligand 1* (*Cxcl1*), *C-C motif chemokine ligand 2* (*Ccl2*), *allograft inflammatory factor 1* (*Aif1*), *glial fibrillary acidic protein* (*Gfap*), *complement component 3* (*C3*), *complement factor B* (*Cfb*), *complement factor H* (*Cfh*), *complement component 1q alpha* (*C1qa*), *complement component 3a receptor 1* (*C3ar1*) and *complement component 5a receptor 1* (*C5ar1*), in the neural retina of light-exposed *Abca4^-/-^ Rdh8^-/-^* mice (**Figure S9**).

Iron dyshomeostasis is a key contributor to retinal degeneration^45^,^46^. Dry AMD patients exhibit significantly elevated retinal iron levels compared to healthy individuals^47^. Gene-set enrichment analysis (GSEA) revealed a positive correlation in genes associated with iron dyshomeostasis in neural retina of dry AMD patients with geographic atrophy compared to age-matched controls (**Figure S10**). Moreover, iron overload induced by intravitreal injection of ferric ammonium citrate increased autofluorescence and triggered geographic atrophy in the retina of mice, which is similar to the retinal changes observed in dry AMD^48^. The accumulation of atRAL in the photoreceptors is considered as a key causative factor of dry AMD^49^. Evidence from our previous studies have shown that atRAL disrupts iron homeostasis to increase iron levels in photoreceptor cells^16^,^17^. In the current study, we demonstrated that GSDME deletion and MitoTEMPO treatment both restored the mRNA levels of *Tfrc*, *Tf* and *HO-1*genes related to iron homeostasis in the neural retina of *Abca4^-/-^Rdh8^-/-^* mice after light exposure (**Figure 1G-1I** and **Figure 6G-6I**). Cell-based assays also confirmed that GSDME knockout and MitoTEMPO treatment both repressed the expression of iron homeostasis-related genes (*Tfrc*, *Tf*, *Ireb2, Fth1, Ftl1* and* Fpn*) and reduced intracellular ferrous iron levels in atRAL-loaded 661W cells (**Figure 2D-2F** and **Figure 5C-5E**). In addition, the western blotting results of nuclear-cytoplasmic fractionation showed that GSDME-N did not enter the cell nucleus to directly regulate iron homeostasis-related genes (**Figure S11**). Taken together, these data imply that GSDME activation and increased mitoROS production incite photoreceptor iron dyshomeostasis caused by atRAL.

Our previous report reveals that atRAL activates GSDME via active caspase-3 and that GSDME-N localizes to mitochondria in 661W cells treated for 6 h with 5 µM atRAL^32^. In the current study, we found that the cleavage of caspase-3 and GSDME was absent in 661W cells exposed to 5 µM atRAL for 3 h; however, protein levels of cleaved caspase-3 and GSDME-N were markedly elevated after 6 h of atRAL treatment (**Figure S12**). This suggests that caspase-3 activation by atRAL involves GSDME cleavage. Additionally, co-immunofluorescence staining of GSDME-N with the mitochondrial marker TOM20 demonstrated mitochondrial accumulation of GSDME-N (**Figure S13**).

Photoreceptor atrophy is the primary cause of vision loss in STGD1 and dry AMD^50^,^51^. Bright light exposure rapidly disrupts the visual cycle of *Abca4^-/-^Rdh8^-/-^* mice, which leads to the accumulation of atRAL in the photoreceptors and subsequent photoreceptor degeneration^9^. In this study, light-exposed *Abca4^-/-^Rdh8^-/-^Gsdme^-/-^* mice exhibited significantly improved retinal function and reduced retinal degeneration compared to light-exposed* Abca4^-/-^Rdh8^-/-^* mice, as evidenced by recovery of the ERG a-wave and b-wave amplitudes, increased retinal thickness and photoreceptor ONL thickness, and reduced fundus lesions and RPE tight junction disruption (**Figure 1A-1F**). We have previously revealed that photoreceptor atrophy caused by atRAL involves ferroptosis^16^. The current investigation showed that eliminating GSDME clearly enhanced the resistance of photoreceptors to atRAL-driven ferroptosis via reducing iron overload and lipid peroxidation (**Figure 1G-1J** and** Figure 2B-2H**), which was achieved by repressing mitoROS generation (**Figure 3**). Indeed, treatment with MitoTEMPO also visibly ameliorated retinal function and structure in light-exposed *Abca4^-/-^Rdh8^-/-^* mice (**Figure 6A-6F**). Evidently, these results highlight the importance of the GSDME/mitoROS axis in photoreceptor ferroptosis and retinal degeneration induced by atRAL.

This study has several limitations. First, although we establish the *Abca4^-/-^Rdh8^-/-^Gsdme^-/-^* mouse model, the lack of data on the cell-specific mechanisms of GSDME and the absence of in vivo rescue experiments (e.g., AAV-mediated photoreceptor GSDME silencing or cell-specific knockout models) preclude the analysis of causal relationships between GSDME and ferroptosis. Second, we validate the therapeutic potential of MitoTEMPO in the atRAL-driven retinal atrophy model, but the pharmacokinetics of MitoTEMPO in the retina need to be analyzed to further refine the therapeutic regimen.

**Figure 7:**
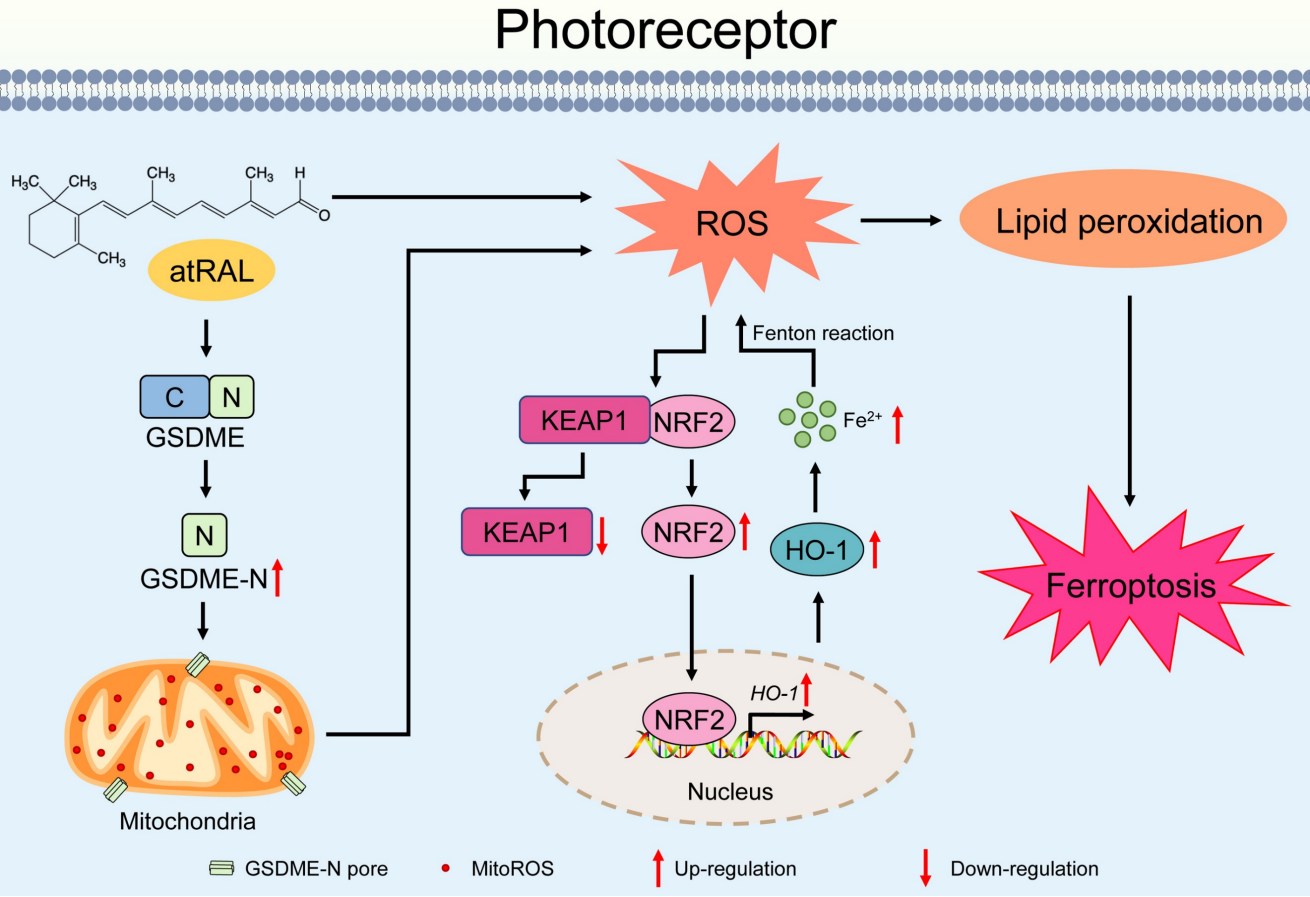
** Proposed mechanisms by which the activation of the GSDME/mitoROS/KEAP1/NRF2/HO-1 axis by atRAL stimulates photoreceptor ferroptosis**.

In summary, our data showed that the activation of the GSDME/mitoROS/KEAP1/NRF2/HO-1 axis by atRAL increases sensitivity to photoreceptor ferroptosis. A plausible mechanism for this discovery was depicted in **Figure 7**. Activation of GSDME by atRAL disrupts the mitochondria, resulting in the generation of mitoROS. These mitoROS trigger oxidative stress by activating the KEAP1/NRF2/HO-1 signaling pathway, causing iron overload and lipid peroxidation, which ultimately lead to ferroptotic cell death. To our knowledge, the present study is the first to report the anti-ferroptotic effect of GSDME deletion on photoreceptors exposed to atRAL, and its underlying mechanism. This work not only deepens our understanding of the pathogenesis of STGD1 and dry AMD but also offers potential avenues for developing novel molecular targeted therapies against retinal degeneration caused by atRAL.

## Supplementary Material

Supplementary figures and tables.

## References

[1] von Lintig J, Moon J, Babino D (2021). Molecular components affecting ocular carotenoid and retinoid homeostasis. Prog Retin Eye Res.

[2] Liu X, Chen J, Liu Z, Li J, Yao K, Wu Y (2016). Potential Therapeutic Agents Against Retinal Diseases Caused by Aberrant Metabolism of Retinoids. Invest Ophthalmol Visual Sci.

[3] Hofmann KP, Lamb TD (2023). Rhodopsin, light-sensor of vision. Prog Retin Eye Res.

[4] Molday RS, Garces FA, Scortecci JF, Molday LL (2022). Structure and function of ABCA4 and its role in the visual cycle and Stargardt macular degeneration. Prog Retin Eye Res.

[5] Xie T, Zhang Z, Fang Q, Du B, Gong X (2021). Structural basis of substrate recognition and translocation by human ABCA4. Nat Commun.

[6] Chen C, Thompson DA, Koutalos Y (2012). Reduction of all-trans-retinal in vertebrate rod photoreceptors requires the combined action of RDH8 and RDH12. J Biol Chem.

[7] Chen Y, Okano K, Maeda T, Chauhan V, Golczak M, Maeda A (2012). Mechanism of all-trans-retinal toxicity with implications for stargardt disease and age-related macular degeneration. J Biol Chem.

[8] Rivera A, White K, Stöhr H, Steiner K, Hemmrich N, Grimm T (2000). A comprehensive survey of sequence variation in the ABCA4 (ABCR) gene in Stargardt disease and age-related macular degeneration. Am J Hum Genet.

[9] Liao C, Cai B, Feng Y, Chen J, Wu Y, Zhuang J (2020). Activation of JNK signaling promotes all-trans-retinal-induced photoreceptor apoptosis in mice. J Biol Chem.

[10] He D, Tao L, Cai B, Chen X, Wang Y, Li S (2023). eIF2alpha incites photoreceptor cell and retina damage by all-trans-retinal. J Biol Chem.

[11] Liang D, Minikes AM, Jiang X (2022). Ferroptosis at the intersection of lipid metabolism and cellular signaling. Mol Cell.

[12] Chen J, Li X, Ge C, Min J, Wang F (2022). The multifaceted role of ferroptosis in liver disease. Cell Death Differ.

[13] Sun S, Shen J, Jiang J, Wang F, Min J (2023). Targeting ferroptosis opens new avenues for the development of novel therapeutics. Signal Transduction Targeted Ther.

[14] Stockwell BR, Friedmann Angeli JP, Bayir H, Bush AI, Conrad M, Dixon SJ (2017). Ferroptosis: A Regulated Cell Death Nexus Linking Metabolism, Redox Biology, and Disease. Cell.

[15] Fang X, Ardehali H, Min J, Wang F (2023). The molecular and metabolic landscape of iron and ferroptosis in cardiovascular disease. Nat Rev Cardiol.

[16] Chen C, Chen J, Wang Y, Liu Z, Wu Y (2021). Ferroptosis drives photoreceptor degeneration in mice with defects in all-trans-retinal clearance. J Biol Chem.

[17] Chen C, Yang K, He D, Yang B, Tao L, Chen J (2023). Induction of ferroptosis by HO-1 contributes to retinal degeneration in mice with defective clearance of all-trans-retinal. Free Radical Biol Med.

[18] Campbell NK, Fitzgerald HK, Dunne A (2021). Regulation of inflammation by the antioxidant haem oxygenase 1. Nat Rev Immunol.

[19] Xiang Q, Zhao Y, Lin J, Jiang S, Li W (2022). The Nrf2 antioxidant defense system in intervertebral disc degeneration: Molecular insights. Exp Mol Med.

[20] McMahon M, Itoh K, Yamamoto M, Hayes JD (2003). Keap1-dependent proteasomal degradation of transcription factor Nrf2 contributes to the negative regulation of antioxidant response element-driven gene expression. J Biol Chem.

[21] Periyasamy P, Shinohara T (2017). Age-related cataracts: Role of unfolded protein response, Ca2+ mobilization, epigenetic DNA modifications, and loss of Nrf2/Keap1 dependent cytoprotection. Prog Retin Eye Res.

[22] Tang Z, Ju Y, Dai X, Ni N, Liu Y, Zhang D (2021). HO-1-mediated ferroptosis as a target for protection against retinal pigment epithelium degeneration. Redox Biol.

[23] Fang X, Wang H, Han D, Xie E, Yang X, Wei J (2019). Ferroptosis as a target for protection against cardiomyopathy. Proc Natl Acad Sci USA.

[24] Privitera G, Rana N, Armuzzi A, Pizarro TT (2023). The gasdermin protein family: emerging roles in gastrointestinal health and disease. Nat Rev Gastroenterol Hepatol.

[25] Zhu C, Xu S, Jiang R, Yu Y, Bian J, Zou Z (2024). The gasdermin family: emerging therapeutic targets in diseases. Signal Transduction Targeted Ther.

[26] Weindel CG, Ellzey LM, Martinez EL, Watson RO, Patrick KL (2023). Gasdermins gone wild: new roles for GSDMs in regulating cellular homeostasis. Trends Cell Biol.

[27] LaRock DL, Johnson AF, Wilde S, Sands JS, Monteiro MP, LaRock CN (2022). Group A Streptococcus induces GSDMA-dependent pyroptosis in keratinocytes. Nature.

[28] De Schutter E, Roelandt R, Riquet FB, Van Camp G, Wullaert A, Vandenabeele P (2021). Punching Holes in Cellular Membranes: Biology and Evolution of Gasdermins. Trends Cell Biol.

[29] Aglietti RA, Dueber EC (2017). Recent Insights into the Molecular Mechanisms Underlying Pyroptosis and Gasdermin Family Functions. Trends Immunol.

[30] Miao R, Jiang C, Chang WY, Zhang H, An J, Ho F (2023). Gasdermin D permeabilization of mitochondrial inner and outer membranes accelerates and enhances pyroptosis. Immunity.

[31] Huang LS, Hong Z, Wu W, Xiong S, Zhong M, Gao X (2020). mtDNA Activates cGAS Signaling and Suppresses the YAP-Mediated Endothelial Cell Proliferation Program to Promote Inflammatory Injury. Immunity.

[32] Cai B, Liao C, He D, Chen J, Han J, Lu J (2022). Gasdermin E mediates photoreceptor damage by all-trans-retinal in the mouse retina. J Biol Chem.

[33] Yang B, Yang K, Xi R, Li S, Chen J, Wu Y (2024). Inhibition of JNK signaling attenuates photoreceptor ferroptosis caused by all-trans-retinal. Free Radical Biol Med.

[34] Chen M, Zhang Y, Jiang K, Wang W, Feng H, Zhen R (2022). Grab regulates transferrin receptor recycling and iron uptake in developing erythroblasts. Blood.

[35] Galy B, Conrad M, Muckenthaler M (2024). Mechanisms controlling cellular and systemic iron homeostasis. Nat Rev Mol Cell Biol.

[36] Yu Y, Jiang L, Wang H, Shen Z, Cheng Q, Zhang P (2020). Hepatic transferrin plays a role in systemic iron homeostasis and liver ferroptosis. Blood.

[37] Balakrishnan M, Kenworthy AK (2024). Lipid Peroxidation Drives Liquid-Liquid Phase Separation and Disrupts Raft Protein Partitioning in Biological Membranes. J Am Chem Soc.

[38] Yang WS, SriRamaratnam R, Welsch ME, Shimada K, Skouta R, Viswanathan VS (2014). Regulation of ferroptotic cancer cell death by GPX4. Cell.

[39] Zhou X, Liu H, Feng F, Kang G-J, Liu M, Guo Y (2024). Macrophage IL-1β mediates atrial fibrillation risk in diabetic mice. JCI Insight.

[40] Bi X, Wu X, Chen J, Li X, Lin Y, Yu Y (2024). Characterization of ferroptosis-triggered pyroptotic signaling in heart failure. Signal Transduction Targeted Ther.

[41] Martínez-Reyes I, Chandel NS (2020). Mitochondrial TCA cycle metabolites control physiology and disease. Nat Commun.

[42] Mukherjee AG, Mishra S, Gopalakrishnan AV, Kannampuzha S, Murali R, Wanjari UR (2025). Unraveling the mystery of citrate transporters in Alzheimer's disease: An updated review. Ageing Res Rev.

[43] Lu X, Jin P, Tang Q, Zhou M, Xu H, Su C (2025). NAD+ Metabolism Reprogramming Drives SIRT1-Dependent Deacetylation Inducing PD-L1 Nuclear Localization in Cervical Cancer. Adv Sci (Weinh).

[44] Liu Q, Zhang L, Chen Z, He Y, Huang Y, Qiu C (2024). Metabolic Profiling of Cochlear Organoids Identifies α-Ketoglutarate and NAD+ as Limiting Factors for Hair Cell Reprogramming. Adv Sci (Weinh).

[45] Xu Y, Huang S, Zhou S, Wang X, Wei M, Chen X (2024). Iron Chelator Deferiprone Restores Iron Homeostasis and Inhibits Retinal Neovascularization in Experimental Neovascular Age-Related Macular Degeneration. Invest Ophthalmol Visual Sci.

[46] Zhang KR, Baumann B, Song Y, Sterling J, Erler EA, Guttha S (2022). Conditional knockout of hephaestin in the neural retina disrupts retinal iron homeostasis. Exp Eye Res.

[47] Biesemeier A, Yoeruek E, Eibl O, Schraermeyer U (2015). Iron accumulation in Bruch's membrane and melanosomes of donor eyes with age-related macular degeneration. Exp Eye Res.

[48] Liu Y, Bell BA, Song Y, Kim HJ, Sterling JK, Kim BJ (2021). Intraocular iron injection induces oxidative stress followed by elements of geographic atrophy and sympathetic ophthalmia. Aging Cell.

[49] Getter T, Suh S, Hoang T, Handa JT, Dong Z, Ma X (2019). The selective estrogen receptor modulator raloxifene mitigates the effect of all-trans-retinal toxicity in photoreceptor degeneration. J Biol Chem.

[50] Romano F, Lamanna F, Boon CJF, Siligato A, Kalra G, Agarwal A (2024). Clinical, Genotypic, and Imaging Characterization of the Spectrum of ABCA4 Retinopathies. Ophthalmol Retina.

[51] Wei W, Anantharanjit R, Patel RP, Cordeiro MF (2023). Detection of macular atrophy in age-related macular degeneration aided by artificial intelligence. Expert Rev Mol Diagn.

