# Supplementary material for: Activation of GSDME by all-trans-retinal increases sensitivity to photoreceptor ferroptosis
Source: Int J Biol Sci. 2025 Oct 27;21(15):7029–42. doi: 10.7150/ijbs.114187 (PMC12631243; doi:10.7150/ijbs.114187)
Supplement: Supplementary file 1 — Supplementary figures and tables. [file ijbsv21p7029s1.pdf]

Supplementary Materials for

Activation of GSDME by all-*trans*-retinal increases sensitivity to photoreceptor ferroptosis

Bo Yang, Kunhuan Yang, Yuling Chen, Ruitong Xi, Jiahuai Han, Shiying Li, Jingmeng Chen, Yalin Wu*

*Correspondence author: Yalin Wu, Xiamen Eye Center and Eye Institute of Xiamen University, School of Medicine, Xiamen University, No. 336 Xiahe Road, Siming District, Xiamen, Fujian 361003, China. E-mail address: yalinw@xmu.edu.cn (Y. Wu).

This file includes:

Supplementary Table S1 to Table S2
Supplementary Figure S1 to Figure S13

Supplementary Table S1. Detailed reagents and antibodies.

Reagents	Catalog	Company
MitoTEMPO	SML0737	Sigma-Aldrich
All-*trans*-retinal (atRAL)	R2500	Sigma-Aldrich
Ferrostatin-1 (Fer-1)	SML0583	Sigma-Aldrich
Hochest 33342	B2261	Sigma-Aldrich
4′,6-diamidino-2-phenylindole (DAPI)	F6057	Sigma-Aldrich
RIPA buffer	R0278	Sigma-Aldrich
Dimethyl sulfoxide (DMSO)	D8371	Solarbio
Rhodamine-123	R302	ThermoFisher Scientific
MitoSOX™ Red mitochondrial superoxide indicator	36008	ThermoFisher Scientific
CellROX™ Deep Red	C10491	ThermoFisher Scientific
Image-iT™ Lipid Peroxidation Kit	C10445	ThermoFisher Scientific
Protease & Phosphatase inhibitors	78442	ThermoFisher Scientific
BCA Protein Assay Kit	23227	ThermoFisher Scientific
BODIPY 581/591 C11	D3861	ThermoFisher Scientific
Pierce Crosslink Magnetic IP/Co-IP Kit	88805	ThermoFisher Scientific
NE-PER Nuclear and Cytoplasmic Extraction Reagents	78833	ThermoFisher Scientific
FeRhoNox-1	GC901	Goryo Chemical
FerroOrange	F374	Dojindo
Anti-COX2	12282S	Cell Signaling Technology
Anti-HO-1	82206S	Cell Signaling Technology
Anti-KEAP1	8047S	Cell Signaling Technology
Anti-NRF2	12721S	Cell Signaling Technology
Anti-GAPDH	5174S	Cell Signaling Technology
Anti-GSDME	ab215191	Abcam
Anti-4-HNE	ab48506	Abcam
Lipofectamine® LTX & PLUS™ reagent	15338100	Invitrogen
Alexa Fluor 594-conjugated donkey anti-mouse secondary antibody	A21203	Invitrogen
Alexa Fluor 594-conjugated donkey anti-rabbit secondary antibody	A21207	Invitrogen
Alexa Fluor 488-conjugated donkey anti-mouse secondary antibody	A21202	Invitrogen
Goat anti-rabbit IgG (H + L) secondary antibody	31460	Invitrogen
TRIeasy total RNA extraction reagent	D606ES60	Yeasen
ReverTra Ace qPCR RT Master Mix	ESQ-201	Toyobo

FastStart Essential DNA Green Master	6402712001	Roche
MTS Assay Kit	G3580	Promega
Amplex Red Citrate Assay Kit	S0335S	Beyotime
Amplex Red α-Ketoglutarate Assay Kit	S0323S	Beyotime
RPE65 Rabbit mAb	A9615	ABclona
TOM20 Rabbit mAb	A19403	ABclona
TSA Fluorescence Triple Staining Kit	RK05903-10	ABclona

Supplementary Table S2. Primer sequences.

Gene	Forward primer (5′→3′)	Reverse primer (5′→3′)
Ptgs2	AATGTATGAGCACAGGATTTGACC	TGTCAGCACATATTTCATGATTAA ACTTCG
HO-1	GGAAATCATCCCTTGCACGC	CCTGAGAGGTCACCCAGGTA
Fpn	GTCTCTGTCAGCCTGCTGTT	CTTGCAGCAACTGTGTCACC
Fth1	CGGGCCTCCTACACCTACCT	CCCTCCAGAGCCACGTCAT
Ftl1	GGAGCGTCTCCTCGAGTTTC	CAGGGCATGCAGATCCAAGA
Ireb2	GTGACACTGTCTCTGTTCGT	TGTGTAACCATCCCACTGCC
Tf	CCAGAGGGTACCACACCTGA	TCCAGGAGTCGTGAGGTTGA
Tfrc	CTCAGTTTCCGCCATCTCAGT	GCAGCTCTTGAGATTGTTTGCA
Gapdh	AGGTCGGTGTGAACGGATTTG	TGTAGACCATGTAGTTGAGGTCA
IL6	TAGTCCTTCCTACCCCAATTTCC	TTGGTCCTTAGCCACTCCTTC
Tnf	CAGGCGGTGCCTATGTCTC	CGATCACCCCGAAGTTCAGTAG
Cxcl1	ACTGCACCCAAACCGAAGTC	TGGGGACACCTTTTAGCATCTT
Ccl2	TTAAAAACCTGGATCGGAACCAA	GCATTAGCTTCAGATTTACGGGT
Aif1	ATCAACAAGCAATTCCTCGATGA	CAGCATTCGCTTCAAGGACATA
Gfap	ACCAGCTTACGGCCAACAG	CCAGCGATTCAACCTTTCTCT
C3	CAGCTTCAGGGTCCCAGCTA	CTCCAGCCGTAGGACATTGG
Cfb	GAGCGCAACTCCAGTGCTT	GAGGGACATAGGTACTCCAGG
Cfh	AGGCTCGTGGTCAGAACAAC	GTTAGACGCCACCCATTTTCC
C1qa	GGGCTCTTTCAGGTGTTAGCA	CGGGGTCCTTTTCGATCCA
C3ar1	TCGATGCTGACACCAATTCAA	TCCCAATAGACAAGTGAGACCAA
C5ar1	ATGGACCCCATAGATAACAGCA	GAGTAGATGATAAGGGCTGCAAC
Glut1	AGCAGCAAGACCGATGAACA	TAGCCGAACTGCAGTGATCC
Ldha	AACTTGGCGCTCTACTTGCT	TAGCCGCCTGAGGACTTACT
Hk2	CTGCTTTGGAGATCCGAGGG	GTCTAGCTGCTTAGCGTCCC
Pkm2	GCAGCGACTCGTCTTCACTT	TCGGCATGGTTCCTGAAGTC
Mpc1	ATGTCCGGAGCAAGGACTTC	AGAAGTGCATCTACCGTGGG
Idh3a	GAGTACGCTCGGAACAACCA	AGTTCTCCGCAACTTCCCTG
Idh3b	GTCACTCGCACCAAGTCTCA	TGTTGGCTTTATGGACGGCT
Sarm1	CCGTGATAAGCAGTGGGGAA	GACCCTGAGTTCCTCCGGTA
Nampt	TGGGGTGAAGACCTGAGACA	TGGCAGCAACTTGTAGCCTT
Nmnat1	CCGAGGTCCTAGGAGACGAG	CCCCAGGGACCGTTACAAAA

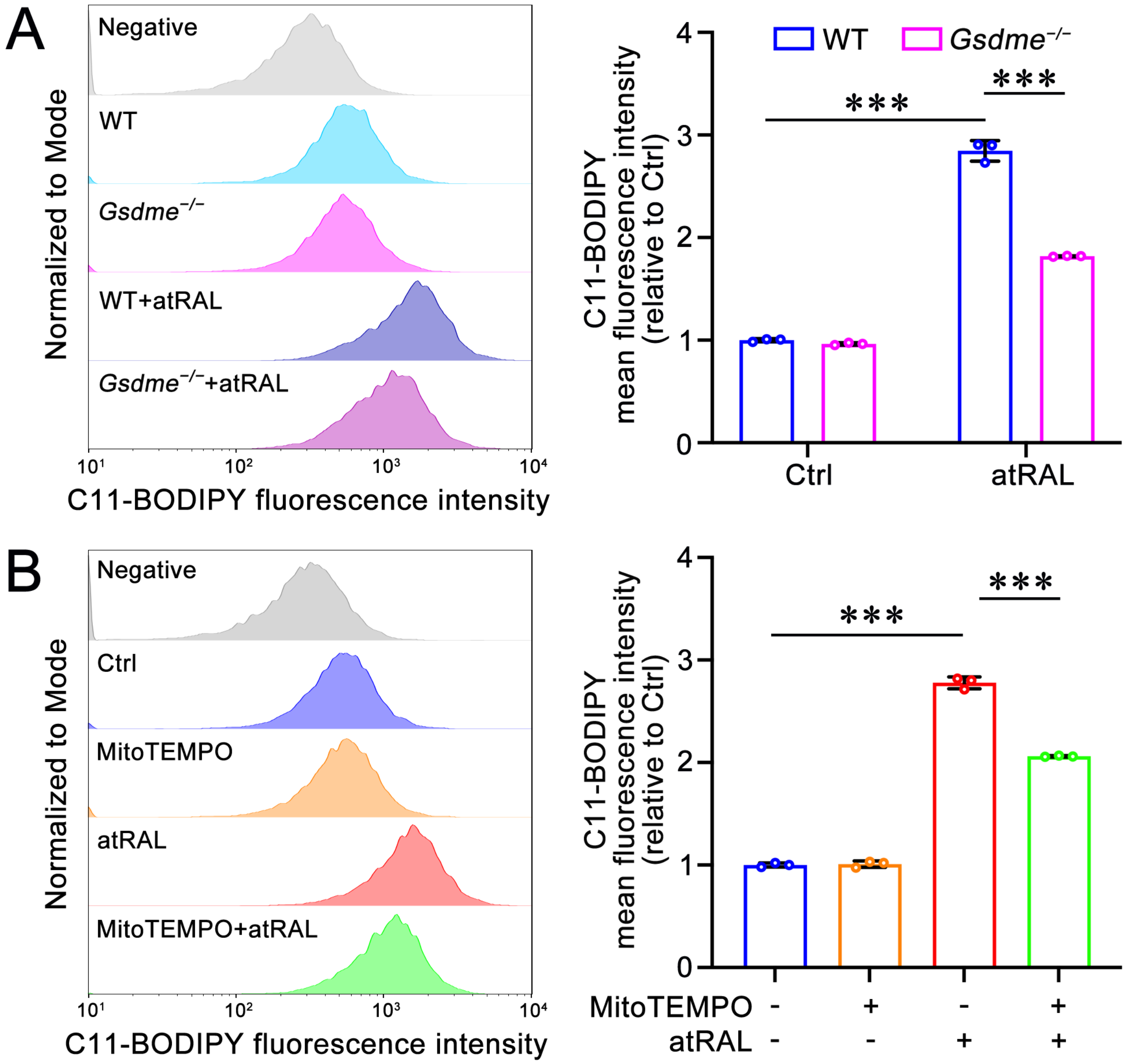

Supplementary Figure S1. Flow cytometric analysis of lipid peroxidation at the cellular level. Lipid peroxidation was measured using C11-BODIPY staining coupled with flow cytometry. (A) WT or *Gsdme*$^{-/-}$ 661W cells were incubated with 5 μM atRAL for 6 h. (B) 661W cells were pretreated with 50 μM MitoTEMPO for 2 h, followed by exposure to 5 μM atRAL for 6 h. ***$p < 0.001$.

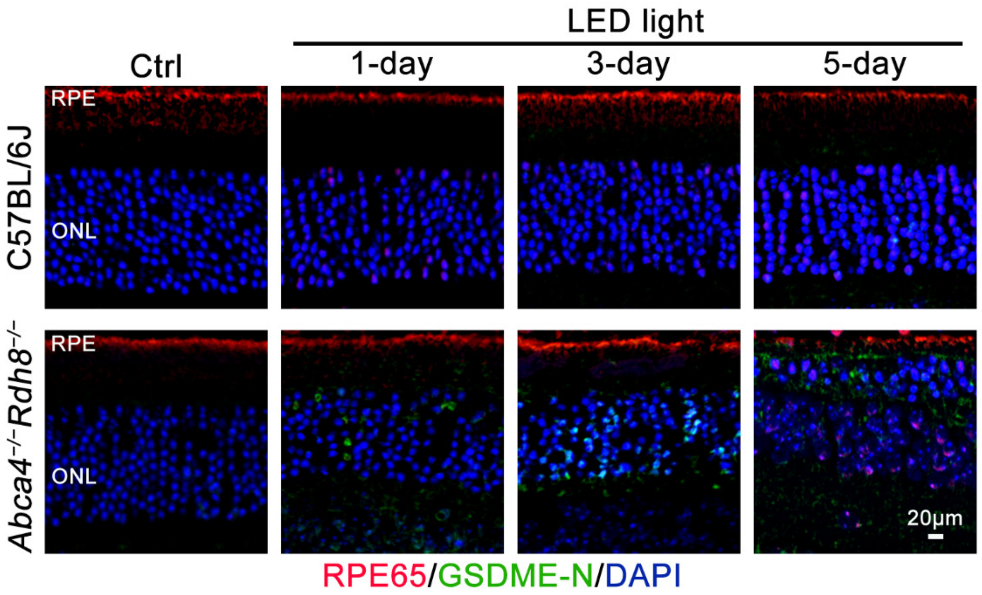

Supplementary Figure S2. GSDME activation is induced and increases over time in photoreceptor ONL of light-exposed *Abca4$^{-/-}$Rdh8$^{-/-}$* mice. C57BL/6J and *Abca4$^{-/-}$Rdh8$^{-/-}$* mice at 4 weeks of age were dark-adapted for 2 days. Following dilation of the pupils with 1% tropicamide, the mice were exposed for 1 h to 10,000-lx LED light and then kept in the dark for 1, 3 and 5 days. Control C57BL/6J and *Abca4$^{-/-}$Rdh8$^{-/-}$* mice were maintained normally in the dark for 7 days without light exposure. Changes in protein levels of GSDME-N in photoreceptor ONL were examined by immunofluorescence staining of mouse retina with an anti-GSDME-N antibody (*green*) and an anti-RPE65 antibody (*red*). Nuclei were stained *blue* with DAPI. Scale bars, 20 µm.

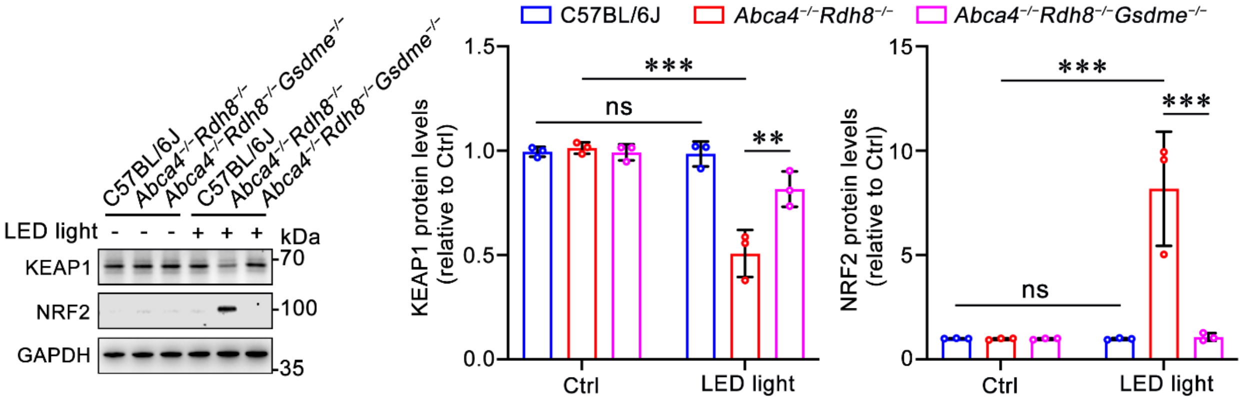

Supplementary Figure S3. GSDME deficiency inhibits light-induced activation of KEAP1/NRF2 signaling in the neural retina of *Abca4$^{-/-}$Rdh8$^{-/-}$* mice. Four-week-old C57BL/6J, *Abca4$^{-/-}$Rdh8$^{-/-}$* and *Abca4$^{-/-}$Rdh8$^{-/-}$Gsdme$^{-/-}$* mice that had been dark-adapted for 2 days were exposed or unexposed to 10,000-lx LED light for 1 h after their pupils were dilated with 1% tropicamide, and then kept in the dark for 5 days. Western blotting analysis and quantification of KEAP1 and NRF2 (n=3). ns, not significant. ***p* < 0.01 and ****p* < 0.001.

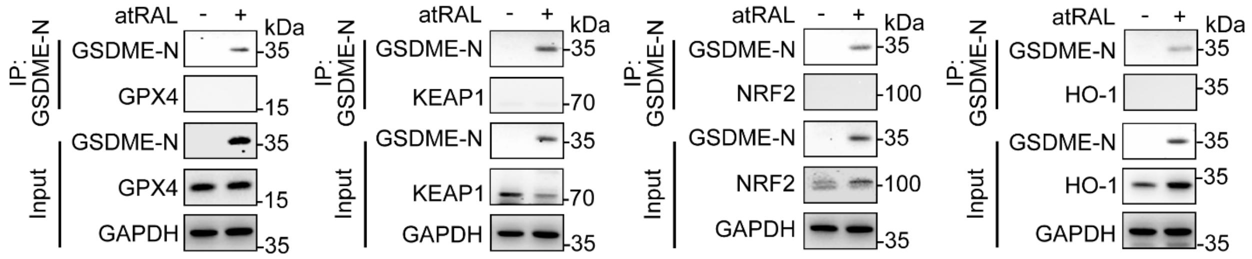

Supplementary Figure S4. Co-immunoprecipitation of GSDME-N and ferroptosis-related key proteins GPX4, KEAP1, NRF2 and HO-1 in atRAL-loaded 661W cells. 661W cells were treated with 5 μM atRAL for 6 h, then harvested and lysed. The cell lysate was subsequently subjected to immunoprecipitation to identify whether ferroptosis-related proteins (GPX4, KEAP1, NRF2 and HO-1) bind to GSDME-N.

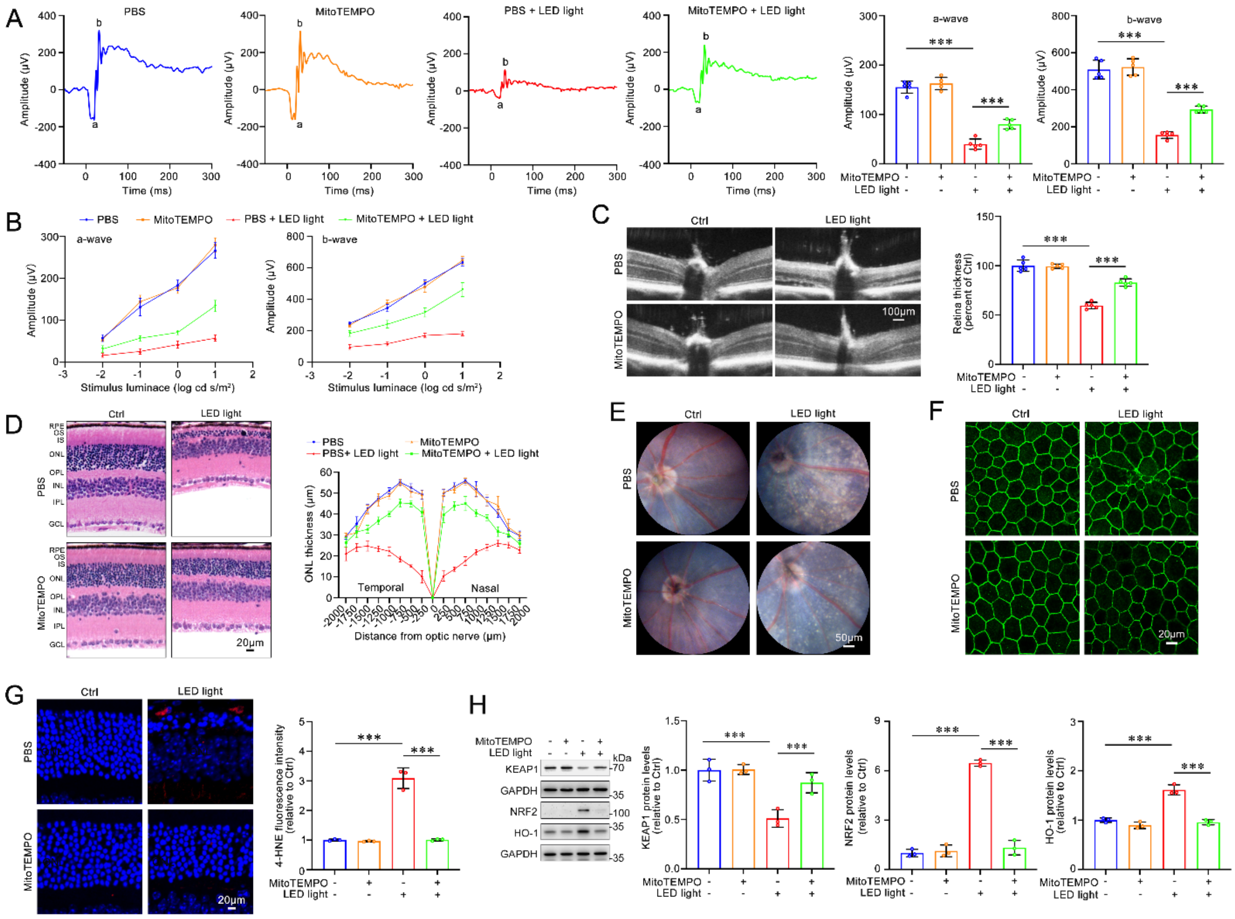

Supplementary Figure S5. Intravitreal injection of MitoTEMPO precludes retinal degeneration and photoreceptor ferroptosis in light-exposed *Abca4*$^{-/-}$*Rdh8*$^{-/-}$ mice. (A) Full-flash ERG, 1 cd s/m^2 (n=6). (B) Full-flash ERG with stimulus luminance levels of 0.01, 0.1, 1 and 10 cd s/m^2 (n=6). (C) Retinal thickness was assessed using OCT. Scale bars, 100 μm. (D) ONL thickness was examined by H&E staining (n=6). Scale bars, 20 μm. (E) Fundus imaging. Scale bars, 50 μm. (F) Whole-mount immunofluorescence staining for the tight junction protein ZO-1 (*green*). Scale bars, 20 μm. (G) 4-HNE levels in the neural retina were quantified by immunofluorescence staining (n=3). Scale bars, 20 μm. (H) Immunoblots and quantification of KEAP1, NRF2 and HO-1 in the neural retina (n=3). ***$p < 0.001$.

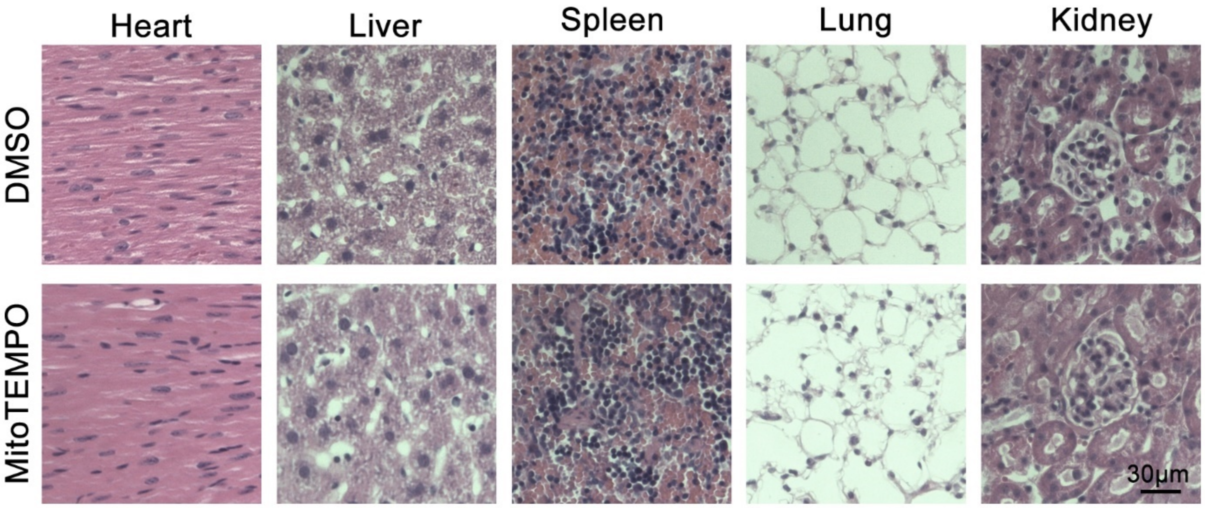

Supplementary Figure S6. The safety of intraperitoneal MitoTEMPO treatment is evaluated in *Abca4$^{-/-}$Rdh8$^{-/-}$* mice. *Abca4$^{-/-}$Rdh8$^{-/-}$* mice aged 4 weeks were intraperitoneally injected once daily with 5 mg/kg MitoTEMPO or DMSO for 5 consecutive days. The systemic safety of MitoTEMPO was then assessed by H&E staining of vital organs (heart, liver, spleen, lungs and kidneys). Scale bars, 30 μm.

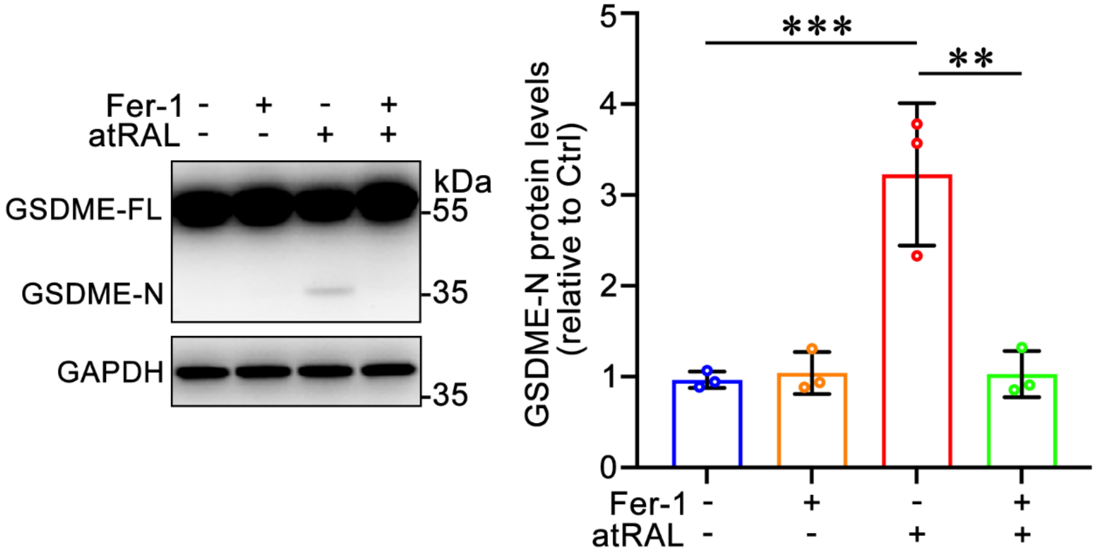

Supplementary Figure S7. Treatment with ferroptosis inhibitor Fer-1 attenuates GSDME activation by atRAL in 661W cells. 661W cells were preincubated with 20 μM Fer-1 for 2 h and then exposed to 5 μM atRAL for 6 h. Immunoblotting analysis of GSDME-FL and GSDME-N, and quantification of GSDME-N (n=3). **$p < 0.01$ and ***$p < 0.001$.

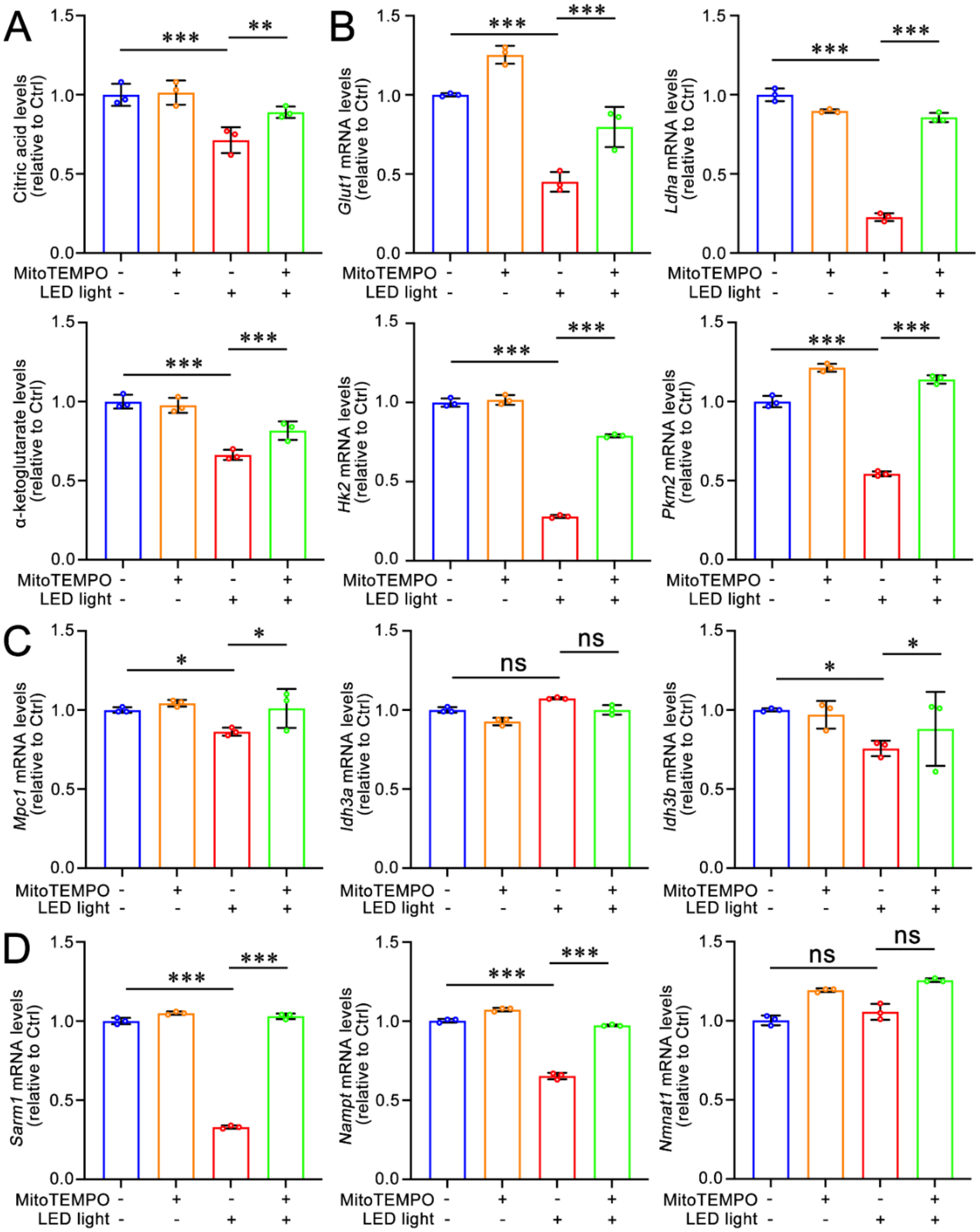

Supplementary Figure S8. Intraperitoneally administered MitoTEMPO restores glycolysis and TCA cycle activity and ameliorates mitochondrial metabolic reprogramming in the neural retina of light-exposed *Abca4*$^{-/-}$*Rdh8*$^{-/-}$ mice. (A) The levels of citric acid and α-ketoglutarate (n=3). (B) The mRNA levels of glycolysis-related genes *Glut1*, *Ldha*, *Hk2* and *Pkm2* (n=3). (C) The mRNA levels of TCA cycle-related genes *Mpc1*, *Idh3a* and *Idh3b* (n=3). (D) The mRNA levels of NAD metabolism-related genes *Sarm1*, *Nampt* and *Nmnat1* (n=3). ns, not significant. **p* < 0.05, ***p* < 0.01, and ****p* < 0.001.

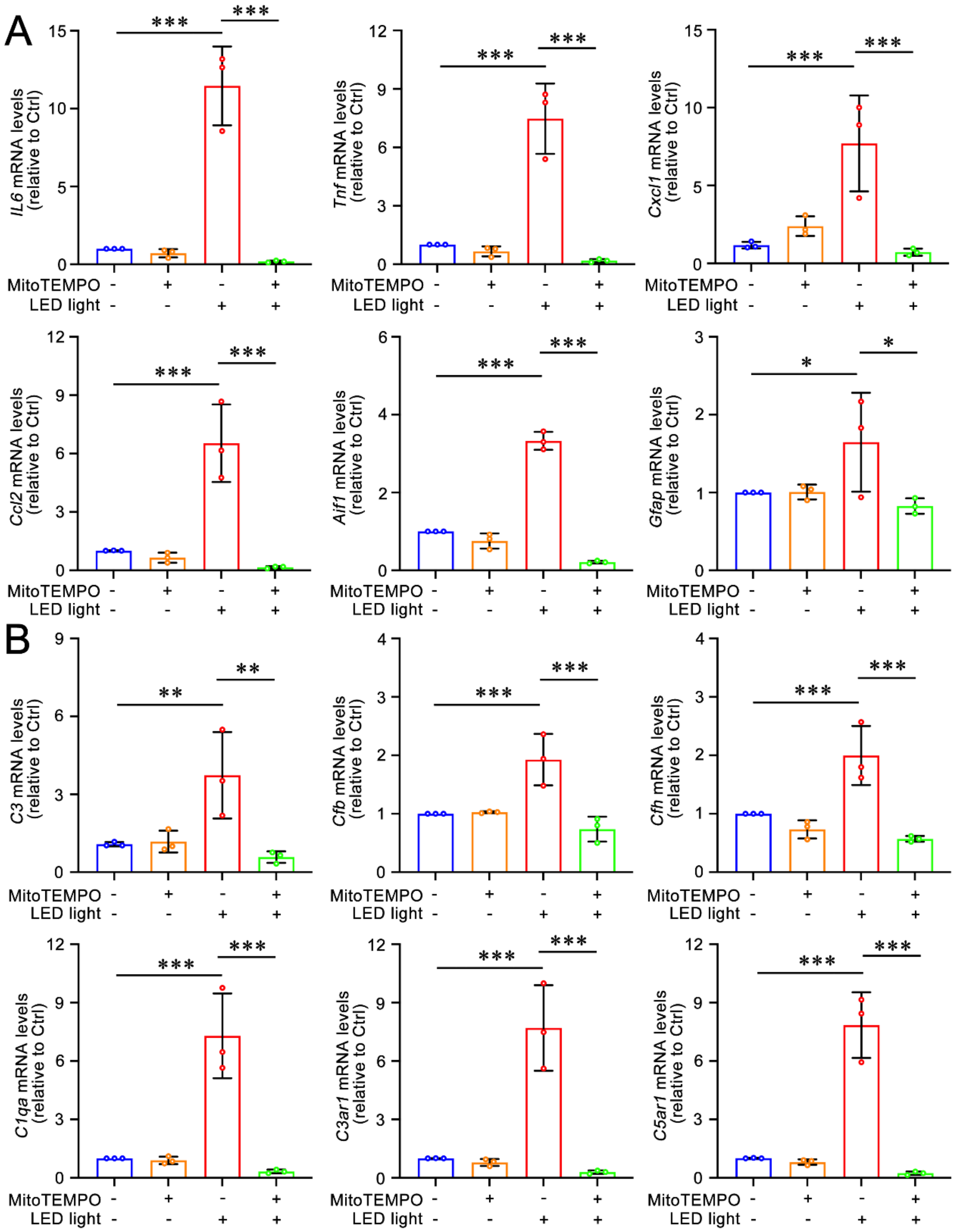

Supplementary Figure S9. Intraperitoneal injection of MitoTEMPO relieves the expression of inflammatory cytokines and complement system components in the retinal microenvironment of light-exposed *Abca4*$^{-/-}$*Rdh8*$^{-/-}$ mice. (A) The mRNA levels of inflammatory cytokines *IL6*, *Tnf*, *Cxcl1*, *Ccl2*, *Aif1* and *Gfap* (n=3). (B) The mRNA levels of complement system components *C3*, *Cfb*, *Cfh*, *C1qa*, *C3ar1* and *C5ar1* (n=3). *$p < 0.05$, **$p < 0.01$ and ***$p < 0.001$.

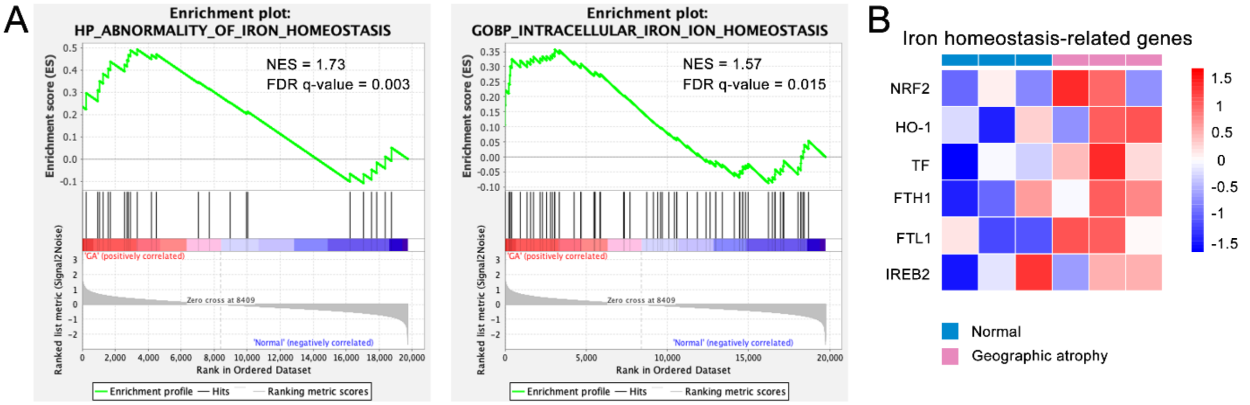

Supplementary Figure S10. The imbalance of iron homeostasis is observed in the neural retina of dry AMD patients with geographic atrophy. (A) GSEA was performed using gene sets 'HP_ABNORMALITY_OF_IRON_HOMEOSTASIS' and 'GOBP_INTRACELLULAR_IRON_ION_HOMEOSTASIS', comparing neural retina of dry AMD patients with geographic atrophy to that of normal controls. (B) A heatmap shows the profiles of iron homeostasis-related genes in neural retina of dry AMD patients with geographic atrophy compared to normal controls.

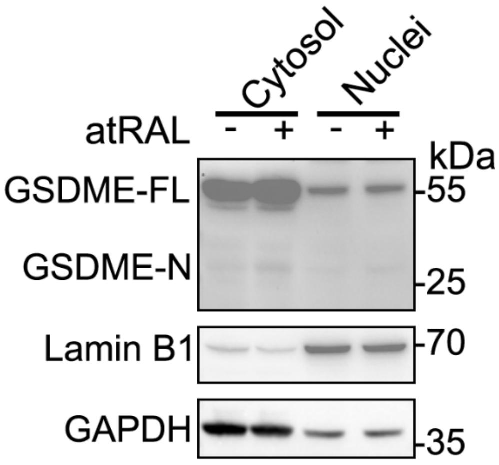

Supplementary Figure S11. **Western blotting was employed to examine the protein levels of GSDME-FL and GSDME-N in the cytosolic and nuclear fractions of 661W cells exposed to atRAL.** Cells were incubated with 5 μM atRAL for 6 h, followed by nuclear-cytoplasmic fractionation. Lamin B1 (nucleus) and GAPDH (cytosol) served as loading controls.

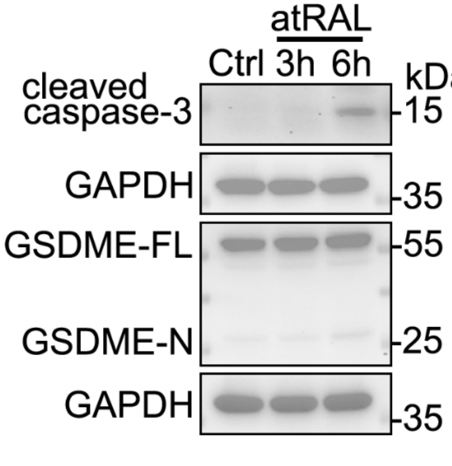

Supplementary Figure S12. **Immunoblotting was used to determine time-dependent protein levels of cleaved caspase-3, GSDME-FL and GSDME-N in 661W cells exposed to atRAL.** Cells were treated with 5 μM atRAL for 3 and 6 h.

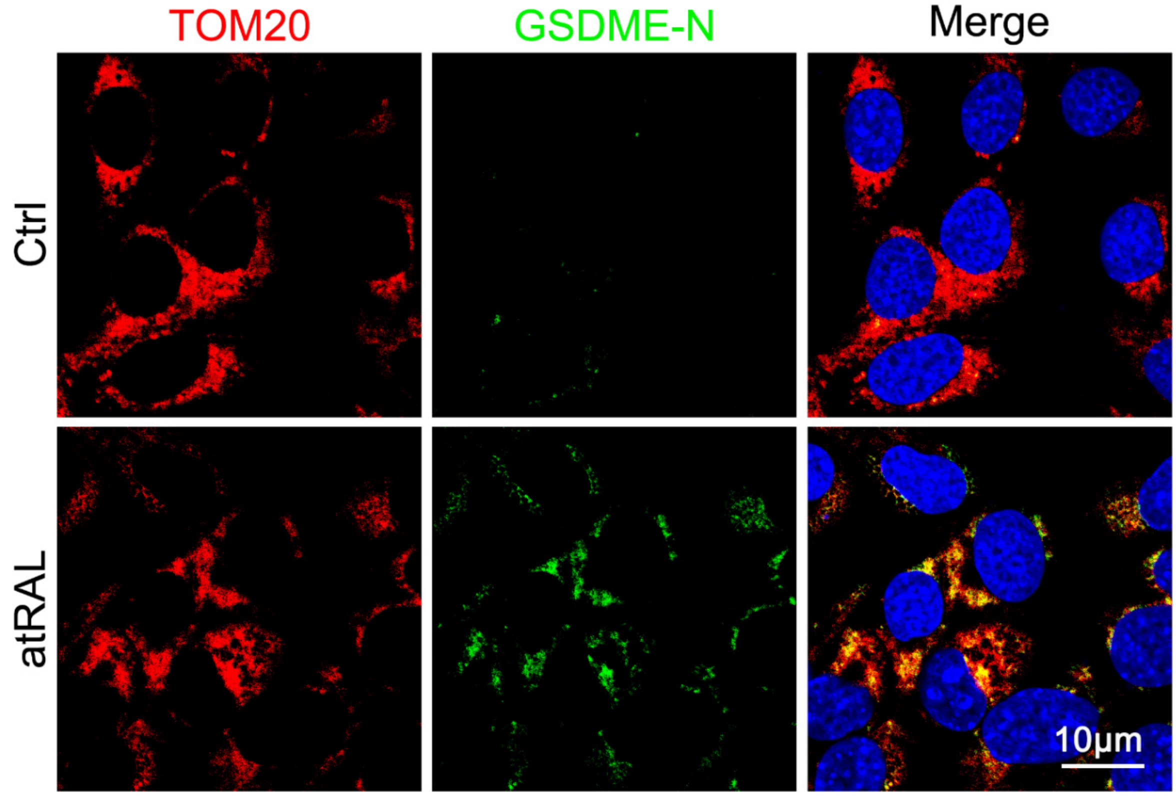

Supplementary Figure S13. **Co-staining of GSDME-N and the mitochondrial marker TOM20 in atRAL-exposed 661W cells.** Cells were incubated with 5 μM atRAL for 6 h. The localization of GSDME-N was assessed by immunofluorescence staining using an anti-GSDME-N antibody (green) and an anti-TOM20 antibody (red). Nuclei were stained blue with DAPI. Scale bars, 10 μm.
